# Hippocampal “cholinergic interneurons” visualized with the choline acetyltransferase promoter: anatomical distribution, intrinsic membrane properties, neurochemical characteristics, and capacity for cholinergic modulation

**DOI:** 10.3389/fnsyn.2015.00004

**Published:** 2015-03-06

**Authors:** Feng Yi, Elizabeth Catudio-Garrett, Robert Gábriel, Marta Wilhelm, Ferenc Erdelyi, Gabor Szabo, Karl Deisseroth, Josh Lawrence

**Affiliations:** ^1^COBRE Center for Structural and Functional Neuroscience, The University of MontanaMissoula, MT, USA; ^2^Department of Biomedical and Pharmaceutical Sciences, The University of MontanaMissoula, MT, USA; ^3^Davidson's Honors College, The University of MontanaMissoula, MT, USA; ^4^Department of Experimental Zoology and Neurobiology, University of PécsPécs, Hungary; ^5^János Szentágothai Research CenterPécs, Hungary; ^6^Department of Sport Biology, University of PécsPécs, Hungary; ^7^Laboratory of Molecular Biology and Genetics, Institute of Experimental Medicine, Hungarian Academy of SciencesBudapest, Hungary; ^8^Department of Bioengineering, Stanford UniversityStanford, CA, USA

**Keywords:** hippocampus, cholinergic modulation, glutamate transmission, optogenetics, transgenic mice

## Abstract

Release of acetylcholine (ACh) in the hippocampus (HC) occurs during exploration, arousal, and learning. Although the medial septum-diagonal band of Broca (MS-DBB) is the major extrinsic source of cholinergic input to the HC, cholinergic neurons intrinsic to the HC also exist but remain poorly understood. Here, ChAT-tauGFP and ChAT-CRE/Rosa26YFP (ChAT-Rosa) mice were examined in HC. The HC of ChAT-tauGFP mice was densely innervated with GFP-positive axons, often accompanied by large GFP-positive structures, some of which were Neurotrace/DAPI-negative and likely represent large axon terminals. In the HC of ChAT-Rosa mice, ChAT-YFP cells were Neurotrace-positive and more abundant in CA3 and dentate gyrus than CA1 with partial overlap with calretinin/VIP. Moreover, an anti-ChAT antibody consistently showed ChAT immunoreactivity in ChAT-YFP cells from MS-DBB but rarely from HC. Furthermore, ChAT-YFP cells from CA1 stratum radiatum/stratum lacunosum moleculare (SR/SLM) exhibited a stuttering firing phenotype but a delayed firing phenotype in stratum pyramidale (SP) of CA3. Input resistance and capacitance were also different between CA1 SR/LM and CA3 SP ChAT-YFP cells. Bath application of ACh increased firing frequency in all ChAT-YFP cells; however, cholinergic modulation was larger in CA1 SR/SLM than CA3 SP ChAT-YFP cells. Finally, CA3 SP ChAT-YFP cells exhibited a wider AP half-width and weaker cholinergic modulation than YFP-negative CA3 pyramidal cells. Consistent with CRE expression in a subpopulation of principal cells, optogenetic stimulation evoked glutamatergic postsynaptic currents in CA1 SR/SLM interneurons. In conclusion, the presence of fluorescently labeled hippocampal cells common to both ChAT-tauGFP and ChAT-Rosa mice are in good agreement with previous reports on the existence of cholinergic interneurons, but both transgenic mouse lines exhibited unexpected anatomical features that departed considerably from earlier observations.

## Introduction

Release of the neurotransmitter acetylcholine (ACh) in the HC is important for learning and memory (Micheau and Marighetto, 2011; Teles-Grilo Ruivo and Mellor, 2013). The major source of acetylcholine in the HC is extrinsic and supplied by the medial septum-diagonal band of Broca (MS-DBB) (Dutar et al., 1995). Lack of HC ACh is associated with cognitive deficits that are observed in Alzheimer's disease (Coyle et al., 1983).

In addition to the MS-DBB cholinergic projection, cholinergic interneurons intrinsic to the hippocampus have been found that may comprise an intrinsic source of ACh (Frotscher et al., 1986, 2000; Freund and Buzsáki, 1996; Romo-Parra et al., 2003). Although originally discovered almost 30 years ago using antibodies to the ACh synthesizing enzyme choline acetyltransferase (ChAT) (Frotscher et al., 1986), no information exists regarding their intrinsic membrane properties. Frotscher and colleagues demonstrated that this population does not contain mRNA for the GABA synthesizing enzymes GAD67 and GAD65 (Frotscher et al., 2000), consistent with the idea that cholinergic interneurons are not GABAergic in nature. With the visualization of these cells using transgenic mouse technology, interest in ChAT-positive cells has resurfaced (von Engelhardt et al., 2007). A recent study in which EGFP was expressed under the control of the ChAT promoter observed that cortical interneurons are highly colocalized with calretinin and/or VIP, implying that cholinergic interneurons are a specialized subpopulation of HC interneurons (Bayraktar et al., 1997; Tricoire and Cea-Del Rio, 2007; von Engelhardt et al., 2007; Chamberland et al., 2010; Chamberland and Topolnik, 2012). GFP-positive neurons have been observed in the HC of transgenic mice in which GFP is driven by the ChAT-promoter (von Engelhardt et al., 2007; Grybko et al., 2011), yet no study has systematically examined this population using transgenic mouse technology in the HC. Here, we investigated fluorescently labeled ChAT-tauGFP and ChAT-YFP neurons in the HC of ChAT-tauGFP and ChAT-Rosa mice, respectively. Consistent with earlier reports, both ChAT-tauGFP and ChAT-YFP neurons labeled a subpopulation of HC neurons in CA1 and CA3 stratum radiatum/stratum lacunosum moleculare. However, we also made several unexpected observations, including cell-sized en passant boutons in ChAT-tauGFP mice and an electrophysiologically distinct subpopulation of CA3 pyramidal cells in ChAT-Rosa mice.

## Material and methods

### Ethics statement

All procedures were performed in accordance with the University of Montana Institutional Animal Care and Use Committee (AUP 017-14).

### Generation of ChAT-Rosa and GAD65-GFP/ChAT-CRE transgenic mice

Rosa26EYFP^+/−^ mice (Soriano, 1999; Srinivas et al., 2001; Madisen et al., 2009) were purchased from Jackson Laboratories (stock no. #007920, Bar Harbor, ME) and bred to homozygosity (Yi et al., 2014). ChAT-CRE mice (GM24 founder line, MMRRC 017269-UCD; Gong et al., 2007; Ivanova et al., 2010) were bred to homozygosity and maintained as a homozygous line. WT, heterozygosity, and homozygosity of ChAT-CRE mice were determined through qPCR similarly to previously established protocols in PV-CRE mice (Tesson et al., 2002; Yi et al., 2014). Heterozygous ChAT-Rosa26EYFP mice were then generated by crossing homozygous ChAT-CRE and homozygous Rosa26EYFP mice. In the present study, ChAT-Rosa was used to refer to ChAT-Rosa26EYFP mouse line, while ChAT-YFP was used to refer to EYFP-positive cells in ChAT-Rosa mice. ChAT-tauGFP mice, in which a tauGFP fusion protein was driven by the ChAT promoter (Grybko et al., 2011), were obtained from Sukumar Vijayaraghavan at University of Colorado-Denver. Homozygous ChAT-CRE mice were crossed with GAD65-GFP mice (López-Bendito et al., 2004; Cea-del Rio et al., 2010). Heterozygous neonates (P1-P3) from this cross were pre-screened for GFP expression using miner's lamp goggles (FHS/F01, Biological Laboratory Equipment Maintenance and Service, Ltd., Budapest, Hungary) equipped with 460-495 nm light source (FHS/LS-1B) and GFP/YFP emission filters (FHS/EF-3GY2) (Cea-del Rio et al., 2010, 2011). GAD65-GFP/ChAT-CRE mice were then bred to homozygosity through this pre-screening method, combined with the determination of CRE zygosity through qPCR (Tesson et al., 2002; Yi et al., 2014). After wean, mice were socially housed in shoebox-style ventilated cages in gender-specific groups (4–5 littermates per cage). Previous studies with ChAT-CRE or ChAT-Rosa mice have shown a high degree of specificity with endogenous ChAT expression in many brain regions, though not complete co-localization (Gong et al., 2007; Ivanova et al., 2010; Witten et al., 2010; Lopes et al., 2012).

### Immunocytochemistry (ICC) in ChAT-Rosa and ChAT-tauGFP mice

Anti-GFP ICC was conducted to intensify the YFP or tauGFP signal, which reduced the laser power required to obtain high quality images, similarly to previously described in PV-GFP (Cea-del Rio et al., 2010) and PV-Rosa mice (Yi et al., 2014). After mice were deeply anesthetized and non-responsive to toe pinch, mice were first transcardially perfused with 50 ml ice cold 0.1 M phosphate buffered saline (PBS), followed by 40–50 ml of ice cold 4% paraformaldehyde (PFA) in PBS, at an approximate rate of 10 ml/min. After clearing of the liver, the mouse was then decapitated with scissors. The brain was carefully removed from the skull and immersed in 4% PFA overnight. In lateralization experiments, an incision was made on the right side of the cortex to preserve orientation in subsequent experiments. On the following day, the tissue block was mounted on a vibratome stage against a block of agarose (4% in dH_2_O) in a PBS bath and sectioned (50 μm thickness) using a vibrating blade microtome (VT1000 S, Leica Microsystems Inc., Buffalo Grove, IL USA). Coronal sections, collected between −1.34 and −2.30 mm from bregma, were collected sequentially in a 24 well plate containing 1 ml PBS per well. For anti-GFP staining, every other slice was chosen, yielding approximately 9–12 slices (each containing a left and right hippocampus) per mouse. On Day 1, HC slices were washed 3 times for 10 min in PBS. Slices were placed in 1 ml of antibody diluent (1% BSA, 0.1% sodium azide, and 0.3% Triton-X in PBS; Gábriel et al., 1992). Primary chicken anti-GFP antibody (directed against YFP; 1:4000, cat# GFP-1020, Aves Labs, Tigard, OR) was then added and left overnight on a shaker at 16°C. In a subset of experiments, goat anti-calretinin antibody (1:200; cat# CG1, Swant, Switzerland) and rabbit anti-VIP antibody (1:200; cat# 9535-0204, AbD Serotec, NC, US) were added to the antibody diluent. On Day 2, slices were washed 3 times in PBS for 10 min each. Slices were then placed in PBS containing secondary antibodies for 2–4 h, followed by 3 washes in PBS for 10 min each. Secondary antibodies included donkey anti-chicken Alexa 488 (1:500; cat# 703-545-155, Jackson ImmunoResearch), donkey anti-goat 647 (1:250, cat# 705-605-143, Jackson ImmunoResearch, West Grove, PA, US), and donkey anti-rabbit Alexa 555 (1:250, cat# A-31572, Life Technologies).

For quantification experiments in ChAT-tauGFP and ChAT-Rosa mice, to define HC layers and label neurons, slices were counterstained with Neurotrace 435/455 Blue Fluorescent Nissl Stain (1:100, cat# N-21479, Life Technologies, Grand Island, NY) or Neurotrace 640/660 (1:100 cat# N-21483, Life Technologies,) for 30–45 min. Slices were then mounted on ColorFrost Plus microscope slides (cat# 9991011, Thermo Scientific) with VectaShield Hardset Mounting Medium (cat # H-1400, Vector Laboratories, Inc.) or Vectashiled Hardset Mounting Medium with DAPI (H-1200, Vector Laboratories, Inc.). Neurotrace 640/660 and DAPI staining exhibited consistent co-localization in all HC cells observed.

### Tyramide signal amplitifcation (TSA)

For anti-ChAT ICC, acute coronal MS-DBB and transverse HC slices at 300 μm were obtained (see Brain Slice Preparation section), incubated with colchicine (100 μg/mL, C9754-1G, Sigma-Aldrich, St. Louis, MO) in sucrose based cutting/storage (SBC) solution for 8 h, containing (in mM): 80 NaCl, 2.5 KCl, 24 NaHCO_3_, 0.5 CaCl_2_, 4 MgCl_2_, 1.25 NaH_2_ PO_4_, 25 glucose, 75 sucrose, 1 ascorbic acid, 3 sodium pyruvate, saturated with 95% O_2_/5% CO_2_ (carbogen), pH 7.4. Slices were then fixed with 2% paraformaldehyde (PFA, cat# 15714-S, Electron Microscopy Sciences, Hatfield, PA) for 1 h (von Engelhardt et al., 2007). Slices were then crytoprotected in 30% sucrose solution in PBS overnight. Brain slices were re-sectioned at 60 μm using a freezing sliding microtome (HM430, Thermo Scientific, Waltham, MA, USA). After 2 hours incubation at room temperature in a gelatin-containing PBS solution (0.2% gelatin; PBS-GT) containing 0.25% Triton X-100 and 10% normal donkey serum, re-sectioned slices were incubated with a goat anti-ChAT primary antibody (1:500; cat# AB144P, EMD Millipore, 3 days) followed by overnight incubation with anti-GFP primary antibody (see previous section). Slices were then incubated with donkey anti-chicken Alexa 488 (1:500) and donkey anti-goat HRP (1:500; cat# AB180P, EMD Millipore) for 60 min. After 10 min incubation with a Tyramide Signal Amplification (TSA) Plus Cyanine Kit (cat# NEL745001KT, PerkinElmer, Waltham, MA) applied directly to the slices and 3xPBS washes, slices were stained with Neurotrace 435/455 Blue Fluorescent Nissl Stain for 30–45 min and mounted on slides. For negative controls, slices containing anti-ChAT primary antibody only or anti-goat HRP secondary antibody and TSA only were processed in parallel (Figure S1).

### Image acquisition, cell counting, and statistical analysis

A total of four ChAT-Rosa mice (11–12 weeks of age, 2 males and 2 females) and five ChAT-tauGFP mice (11–12 weeks of age, 2 males and 3 females) were used for quantification. Images were acquired with a Fluoview FV-1000 confocal imaging system equipped with 10× and 60× objectives (Olympus Center Valley, PA). Blue (405 nm), green (488 nm), and/or red (647 nm) channels were acquired sequentially. Tiles were acquired with ~10% overlap per field. Tiles were flat projected, saved as TIFF files, and either stitched automatically (as part of acquisition in Fluoview) or stitched manually (with Image J). HC ChAT-tauGFP or ChAT-YFP structures were scanned and marked with a color-coded symbol to ensure that they were not counted twice. Neurotrace-positive and Neurotrace-negative structures were distinguished in ChAT-tauGFP mice by toggling between channels in Fluoview or ImageJ. ChAT-tauGFP slices labeled with anti-GFP, DAPI, and Neurotrace 640/660 were selected for diameter measurements. In ImageJ, potential ChAT-tauGFP-positive cells were outlined with the polygon tool and added as objects to the ROI manager box. Feret's diameter (maximum caliper) was measured for each object and the results table was transferred to Excel.

Counting was performed in 11 areas: CA3 (SO, SP, SR, and SLM), CA1 (SO, SP, SR, and SLM), dentate gyrus (SM and SG), and hilus, comprising 11 groups. Each group (from 88 total hippocampi in ChAT-Rosa mice or 112 total hippocampi in ChAT-tauGFP mice) represented the total number of cells counted across all hippocampi. Groups were statistically compared on a per slice and per-layer basis using Prism 6 (Graphpad Software, Inc., La Jolla, CA). Most groups failed tests for normality (D'Agostino & Pearson omnibus normality test and Shapiro–Wilk normality test). Therefore, a Friedman test followed by Dunn's multiple comparisons test were used. The dentate granule cell layer and the hilus passed tests for normality; therefore, lateralization data were statistically compared with a One-Way ANOVA and Tukey's multiple comparison's test.

### Stereotaxic injection of ChR2-mCherry AAV into dorsal CA1 HC

AAV9 EF1.DIO.hChR2(H134R)-mCherry.WPRE.hGH (~10^12^ vc/ml) was strereotaxically injected into the ventral CA1 HC of adult GAD65-GFP/ChAT-CRE mice. Equipment and procedures for stereotaxic injection of AAV into dorsal CA1 HC of GAD65-GFP/ChAT-CRE mice were as previously described (Yi et al., 2014). Stereotaxic coordinates for AAV injection into ventral CA1 HC (1.5 μL at 0.25 μL/min per hemisphere) were: AP 2.9 mm, ML 3.3 mm, and DV 2.3 mm. Injected mice were used for imaging or electrophysiological recordings at least 2 weeks after survival surgery.

### Brain slice preparation

Both male and female ChAT-Rosa (24–45 day old) or adult GAD65-GFP/ChAT-CRE mice were used. Mice were anesthetized with 4% isoflurane and transcardially perfused with oxygenated, ice-cold, SBC solution. After decapitation, the brain was immediately placed in SBC solution saturated with carbogen. Transverse HC slices or coronal MS-DBB slices were cut at 300 μm on a Leica 1200S Vibratome, using the Leica Vibrocheck device to minimize vibration of the blade in the z-direction prior to use (Geiger et al., 2002). After sectioning, slices were placed in a storage chamber and incubated with carbogen-saturated SBC solution at 36–37°C until use.

### Whole-cell patch clamp recordings

After incubation for at least 30 min with SBC solution at 36–37°C, a single slice was gently placed on poly-D-lysine-coated glass coverslips (12 mm diameter, 0.09–0.12 thickness, cat# 633009, Carolina Biological Supply Company, Burlington, NC) and perfused at 34–35°C (TC-324B, Warner Instruments, Hamden, CT, USA) with extracellular solution (in mM): 125 NaCl, 2.5 KCl, 25 NaHCO_3_, 2 CaCl_2_, 1 MgCl_2_, 1.25 NaH_2_PO_4_ and 20 glucose, saturated with carbogen, pH 7.4. Acute slices were then viewed using Infrapatch (Luigs and Neumann, Ratingen Germany) on an upright microscope (Axio Examiner D1, Carl Zeiss Microscopy, LLC, USA). Fluorescent YFP+ cells in slices from ChAT-Rosa mice or GAD65-GFP cells were identified using a Zeiss LED (505 nm for YFP; 470 nm for GFP; Colibri, Carl Zeiss Microscopy, LLC, USA) and subsequently viewed under IR-Dodt contrast with a 63× water immersion objective (W Plan-Apo 63x/1.0 VIS-IR WD = 2.1 M27, Carl Zeiss Microscopy, LLC, USA), similarly to previously described (Yi et al., 2014). Thin-wall glass capillaries (TW150F-3, World Precision Instruments; Sarasota, FL) were fabricated with a 2.5–4.5 MΩ tip resistance on a 2-step PC-10 Narishige vertical puller (East Meadow, NY, USA). Whole-cell recordings were obtained using a Multiclamp 700 B amplifier (Molecular Devices, Union City, CA), filtered at 4 kHz, and digitized at 20 kHz (Digidata 1440 A, Molecular Devices). Glass capillaries contained intracellular solution (IC; in mM): 110 potassium gluconate, 40 KCl, 10 HEPES, 0.1 EGTA, 4 MgATP, 0.3 Na_2_GTP, 10 phosphocreatine and 0.2% biocytin, pH 7.2, osmolarity 290–300 mOsm. Seal resistances ranged from 1–2 GΩ and access resistance ranged from 4–20 MΩ. Bridge balance was used throughout current-clamp experiments and was monitored with a 100 ms long hyperpolarizing current step from −60 mV every 20 s. If access resistance changed by >20%, data were excluded from further analysis. For optogenetic stimulation, TTL-driven flashes from Zeiss LED were delivered to the whole slice to excite ChR2-mCherry-positive neurons. The AAV9-ChR2-mCherry infected ChAT-CRE cells in HC were reliably excited (9/9 cells) by delivery of 2 ms 470 nm light pulse at 5 Hz (Figure S2).

### Anatomical identification of recorded cells

Biocytin (0.2%) was included in the IC for *post-hoc* morphological identification of each recorded cell. Whole-cell mode was maintained for at least 15 min, the electrode was withdrawn slowly to allow cell membrane resealing to an outside-out patch, and the slice was perfused for an additional 10–15 min to allow biocytin to diffuse to distal intracellular compartments. Slices were fixed overnight at 4°C in PBS containing 4% PFA, transferred to PBS, and stored for up to 1 week at 4°C. After permeabilization with 0.3% Triton X-100 in PBS for 2 h at room temperature, slices were incubated in PBS overnight at 16°C with Alexa Fluor 633-conjugated streptavidin (cat # S-21375, final concentration 1 μg/ml; Life Technologies, Grand Island, NY) in PBS. Slices were cryopreserved in 30% sucrose containing PBS and resectioned at 100–150 μm thickness using a freezing sliding microtome. Resectioned slices underwent PBS washes, were incubated with Neurotrace 435/455 Blue Fluorescent Nissl Stain (1:100 in PBS) for 20 min, and were mounted on Colorfrost Plus slides (cat #99-910-11, Thermo Scientific) using Vectashield HardSet mounting medium (cat #H-1400, Vector Laboratories, Inc., Burlingame, CA). Sections were imaged with a Fluoview FV-1000 confocal imaging system (Olympus, Center Valley, PA) with a 25× objective (XLPL25XWMP, Olympus, Tokyo, Japan). Confocal stacks (800 × 800 pixels) of recorded cells were flat projected, rotated, and cropped in Photoshop 13.0 for display.

### Chemical reagents

Acetylcholine chloride (A6625), DNQX (D0540), and gabazine (SR-95531; S106) were obtained from Sigma-Aldrich Inc. (St. Louis, MO). DL-APV was obtained from R&D Systems (Minneapolis, MN).

### Analysis of electrophysiological data

Electrophysiological data analysis was performed with Axograph X (Axograph Scientific, Sydney, Australia). All parameters, which included afterdeflection (ADF), input resistance (R_in_) time constant (τ_m_), cellular capacitance (C_m_), action potential (AP) half width (measured from the first AP), and sag ratio (steady state (SS)/peak; in response to a 1 s long, −100 pA current step from −60 mV), were measured as described previously (Yi et al., 2014). A One-Way ANOVA and Tukey's multiple comparison test or a One-Way ANOVA and Dunn's multiple comparison test was used when appropriate.

## Results

### Region- and lamina-specific expression in the HC of ChAT-tauGFP and ChAT-Rosa mice

**Figure 1:**
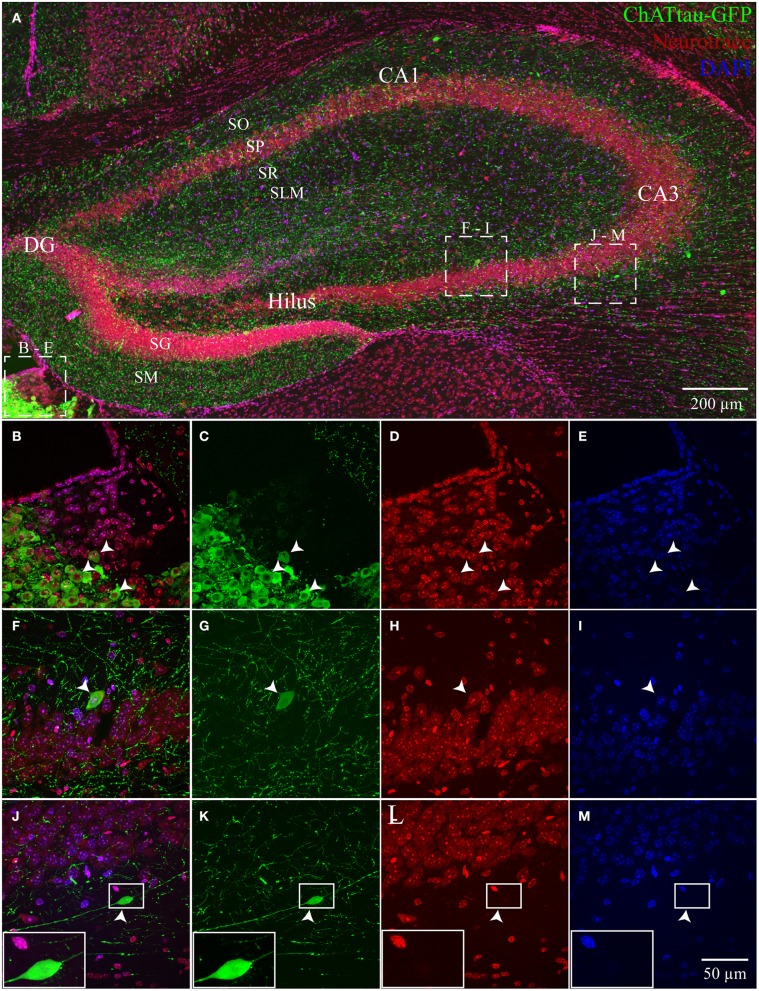
**Hippocampus and habenula in ChAT-tauGFP mice. (A)** Flat-projected confocal image displaying (green) ChAT-tauGFP cells in the hippocampus. Cells and layers are counterstained with (red) Neurotrace 640/660 and DAPI (blue). Representative examples of Neurotrace/DAPI-positive ChAT-tauGFP cells in **(B–E)** habenula and **(F–I)** CA3 SP. **(J–M)** Representative example of a Neurotrace/DAPI-negative ChAT-tauGFP structure in the CA3 SO layer. (J-M, inset) Magnified view suggesting an en passant bouton. Dotted boxes in **(A)** show the relationship to **(B–M)**.

In ChAT-tauGFP mice, consistent with a previous study (Grybko et al., 2011), GFP labeling was highly visible as a dense plexus of axonal processes innervating every layer of the HC (Figure 1A). Occasionally, large, globular structures were observed among ChAT-tauGFP-positive fibers, suggestive of cholinergic interneurons (Grybko et al., 2011). To define HC layers and determine the cellular distribution of ChAT-tauGFP interneurons, sections were counterstained with DAPI and Neurotrace 640/660. As a positive control, ChAT-tauGFP cells were readily observed in the habenula (Figures 1A–E). In the HC, upon closer inspection, a subset of these cells were co-labeled with DAPI and Neurotrace (Figures 1F–I), consistent with a population of HC cholinergic interneurons (Frotscher et al., 1986, 2000; Grybko et al., 2011). Surprisingly, a subset of cell-sized ChAT-tauGFP-positive globular structures were negative for both DAPI and Neurotrace (Figures 1J–M). Axonal fibers were often observed leading into and out of these globular structures, suggesting that the were large, en passant boutons (see also Figures S3E–J).

**Figure 2:**
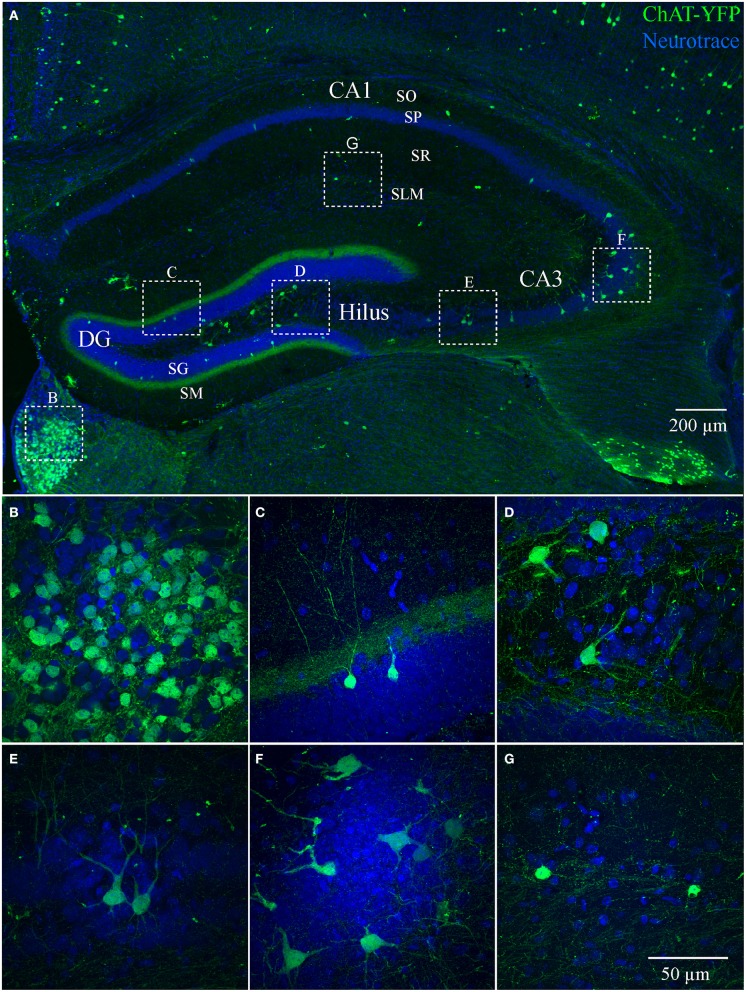
**Hippocampus and habenula in ChAT-Rosa mice. (A)** Flat-projected confocal image displaying ChAT-YFP cells (green) in the hippocampus. Cells and layers are counterstained with Neurotrace 435/455 (blue). Magnified views of ChAT-YFP cells in **(B)** medial habenula and hippocampal subregions **(C)** DG, **(D)** hilus, **(E,F)** CA3, and **(G)** CA1. Dotted boxes in **(A)** show the relationship to **(B–G)**.

We also examined ChAT-Rosa mice to visualize HC ChAT-YFP neurons. In contrast to the prominent axonal labeling in ChAT-tauGFP mice, YFP appeared to be localized predominantly to the somatodendritic domains, possibly due to the limited diffusion of cytosolic YFP into axon terminals. In ChAT-Rosa mice, ChAT-YFP structures in the HC were unambiguously neurons due to dendritic labeling and Neurotrace positivity (Figure 2). In accordance with previous studies (Frotscher et al., 1986, 2000), ChAT-YFP cells were distributed throughout the HC (Figure 2A). Consistent with expected expression of YFP in brain regions known to contain cholinergic neurons, ChAT-YFP cells were observed in the cortex (Figure 2A) (von Engelhardt et al., 2007) and medial habenula (Figures 2A,B) (Grybko et al., 2011; Ren et al., 2011). Within the HC, ChAT-YFP cells were found in the DG (Figures 2A,C), hilus (Figures 2A,D), CA3 (Figures 2A,E,F), and CA1 (Figures 2A,G). The diffuse YFP staining in the DG inner molecular layer, combined with the presence of YFP-positive cells in hilus (Figures 2A,D), suggests that mossy cells are labeled in ChAT-Rosa mice.

**Figure 3:**
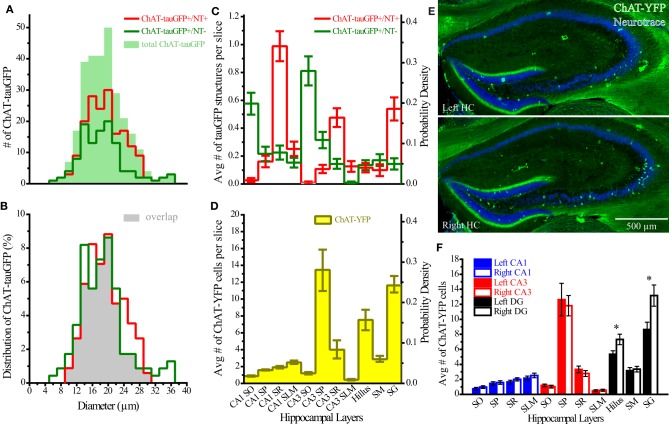
**Quantification of ChAT-tauGFP and ChAT-YFP cells. (A)** Distribution of ChAT-tauGFP diameters (filled green; *n* = 279), categorized by presence (red, indicating true cells, *n* = 167) or absence (green, non-cells, *n* = 116) of Neurotrace 640/660 (2 μm bin). **(B)** Probability density; filled gray indicates the area of overlap. **(C)** Region and layer distribution for ChAT-GFP cells and non-cells. **(D)** Region and layer distribution of ChAT-YFP cells (all were Neurotrace-positive). Panels **(C,D)** are displayed both as average number per slice (left Y axis) and probability density function (right Y axis). **(E)** Flat-projected, confocal images of left (upper, horizontally flipped) and right (lower) HC from a ChAT-Rosa mouse, counterstained with (blue) Neurotrace 435/455. **(F)** Average number of cells in left (solid bars) or right (open bars) hippocampus (31 bilateral hippocampal sections across 3 ChAT-Rosa mice). ^*^Denotes *p* < 0.05.

Theoretically, the distribution of ChAT-tauGFP and ChAT-YFP cells in the HC should be similar. However, due to the initial observation that some ChAT-tauGFP structures were negative for DAPI and Neurotrace (Figures 1J–M), we investigated whether Neurotrace-negative and Neurotrace-positive ChAT-tauGFP structures could be differentiable based on size. Using the Ferret diameter as a measure (Figures 3A,B), size was not significantly different (*p* = 0.16, Mann–Whitney test; from 42 hippocampi of 2 mice) between of DAPI/Neurotrace-positive (18.4 ± 0.4 μm, *n* = 163) and DAPI/Neurotrace-negative structures (18.0 ± 0.6 μm; *n* = 116). These two groups had a similar distribution of diameters (Figure 3A). Moreover, the probability density function revealed a large region of overlap (80.6%; Figure 3B, gray), indicating that ChAT-tauGFP neuronal and non-neuronal structures could not readily be differentiated from each other based on size.

Because DAPI staining confirmed that Neurotrace was a reliable neuronal marker, we pooled quantification from 2 mice counterstained with DAPI/Neurotrace (Figure 1) and 3 mice counterstained with Neurotrace only (Figure S3). Consistent with the cellular distribution observed in ChAT-immunopositive cells in rat (Frotscher et al., 1986, 2000) and ChAT-EGFP cells in mouse (von Engelhardt and colleagues, unpublished observations), we found that the majority of Neurotrace-positive ChAT-tauGFP cells were observed in the stratum radiatum (SR) layer of CA1 (34.1 ± 3.7%, *p* < 0.05) and CA3 (16.4 ± 2.3%, *p* < 0.05), with fewer cells observed in other HC layers (Figure 3C, red; Table 1). Interestingly, the distribution of Neurotrace-negative structures was different, with a high percentage of these structures present in the stratum oriens of CA1 (19.9 ± 2.7%, *p* < 0.05) and CA3 (28.0 ± 4%, *p* < 0.05) (Figure 3C; Table 1). In summary, Neurotrace-positive ChAT-tauGFP cell counts are consistent with earlier work, but our results also reveal cell-sized Neurotrace-negative structures that resembled en passant boutons in ChAT-tauGFP mice.

**Table 1:** **Regional and laminar distribution of ChAT-tauGFP counts**.

**Region**	**GFP+**	**layer**	**GFP+**	**GFP+/NT+**	**GFP+/NT-**
CA1	289	SO	67	3 (0.9%)^†^	64 (19.9%)^*^
		SP	42	18 (5.6%)^†^	24 (7.5%)
		SR	135	110 (34.1%)	25 (7.8%)
		SLM	45	28 (8.7%)^†^	17 (5.3%)
CA3	222	SO	91	1 (0.3%)^†^	90 (28.0%)^*^
		SP	47	12 (3.7%)^†^	35 (10.9%)
		SR	69	53 (16.4%)^§^	16 (5.0%)
		SLM	15	14 (4.3%)^†^	1 (0.3%)
Hilus	28	Hilus	28	13 (4.0%)^†^	15 (4.7%)
DG	106	SM	30	11 (3.4%)^†^	19 (5.9%)
		SG	76	60 (18.6%)^§^	16 (5.0%)

*Values in the table were compiled from 56 hippocampal slices (112 hippocampi total) from 5 ChAT-tauGFP mice*.

† *Denotes p < 0.05 compared to CA1 SR layer among GFP+/NT+ group*.

§ *Denotes p < 0.05 compared to CA3 SO layer among GFP+/NT+ group*.

* *Denotes p < 0.05 compared to CA3 SLM layer among GFP+/NT− group. The percentage numbers are of the fraction of GFP+/NT+ or GFP/NT− within the their groups*.

We also quantified the distribution of ChAT-YFP cells in ChAT-Rosa mice. There was a larger number of ChAT-YFP cells in DG (22.3 ± 1.0, *p* < 0.0001) and CA3 (19.4 ± 1.2, *p* < 0.0001) than CA1 (6.9 ± 0.4, *n* = 88 hippocampi from 4 mice). Layer-specific differences were also observed within the HC of ChAT-Rosa mice (*p* < 0.0001, Friedman test; Figure 3D; Table 2). Within area HC CA1, there were more ChAT-YFP cells in stratum lacunosum moleculare (SLM; 2.5 ± 0.2, *p* < 0.0001) and SR (1.9 ± 1.4, *p* = 0.017) than in SO (0.9 ± 0.1). Within CA3, the region most densely populated with ChAT-YFP cells was in SP (13.7 ± 1.0), compared to SO (1.3 ± 0.1, *p* < 0.0001), SR (4.0 ± 0.3, *p* < 0.0001), and SLM (0.4 ± 0.1, *p* < 0.0001). This high abundance of ChAT-YFP cells in SP was unique to CA3 and not observed in CA1 SP (1.6 ± 1.4, *p* < 0.0001). By contrast, there were more ChAT-YFP cells in CA1 SLM than CA3 SLM (0.4 ± 0.1, *p* < 0.0001). There were also layer-specific differences within DG. Similar to CA3, the highest density of ChAT-YFP cells was observed in the principal cell layer (11.8 ± 0.7) relative to the stratum moleculare layer of the dentate (2.9 ± 0.2, *p* < 0.0001). The hilus (7.6 ± 0.4) also had abundant ChAT-YFP cells comparable to the CA3 SP and granule cell layer of the DG (*p* > 0.05).

**Table 2:** **Regional and laminar distribution of ChAT-tauGFP and ChAT-YFP cells**.

**Region**	**Layer**	**ChAT-tauGFP**	**ChAT-YFP**
CA1	SO	0.03 ± 0.02	0.9 ± 0.1^*^
	SP	0.16 ± 0.04	1.6 ± 0.1^*^
	SR	0.98 ± 0.10	1.9 ± 1.4^*^
	SLM	0.25 ± 0.05	2.5 ± 0.2^*^
CA3	SO	0.01 ± 0.01	1.2 ± 0.1^*^
	SP	0.11 ± 0.03	13.7 ± 1.0^*^
	SR	0.47 ± 0.07	4.0 ± 0.3^*^
	SLM	0.13 ± 0.04	0.4 ± 0.1^*^
Hilus	Hilus	0.12 ± 0.03	7.6 ± 0.4^*^
DG	SM	0.98 ± 0.04	2.9 ± 0.2^*^
	SG	0.54 ± 0.08	11.8 ± 0.7^*^

Data in the table are represented as mean cell number ± SEM per hippocampus, from a total of 44 hippocampal slices (88 hippocampi) from ChAT-Rosa mice and a total of 56 hippocampal slices (112 hippocampi) from ChAT-tauGFP mice. Asterisks denote multiple unpaired t-tests

* *p < 0.05*.

For every HC region, ChAT-YFP cells, which were invariably Neurotrace-positive, were more abundant than Neurotrace-positive ChAT-tauGFP cells (*p* < 0.05, multiple *t*-tests, Table 2). When normalized to total cell number, cellular distribution was different between ChAT-tauGFP and ChAT-Rosa mice. The majority of Neurotrace-positive ChAT-tauGFP cells were located in the in CA1 SR, CA3 SR, and DG SG layer (Figure 3C, red). In contrast, the majority of ChAT-YFP cells were present in CA3 SP, DG SG layer, and hilus (Figure 3D).

Finally, a lateralization difference was detected in the HC of ChAT-Rosa mice. The DG SG layer displayed higher abundance of ChAT-YFP cells in the right (13.2 ± 1.4) than left (8.6 ± 1.0) hemisphere (*p* = 0.001, paired *t*-test, Figures 3E,F). A right preference of ChAT-YFP cells was also observed in the hilus (R: 7.3 ± 0.7; L: 5.4 ± 0.5, *p* = 0.009, paired *t*-test, *n* = 31 HC slices from 3 mice). There was no lateralization bias in CA1 or CA3 (*p* > 0.05). On a per mouse basis, there are the same lateralization trends, although without significant difference, in DG SG layer (R: 136.0 ± 21.0; L: 89.3 ± 7.5, *n* = 3) and hilus (R: 75.7 ± 16.8; L: 55.3 ± 4.8, *n* = 3).

### Anti-ChAT immunoreactivity is more readily detected in ChAT-YFP cells from MS-DBB than HC

**Figure 4:**
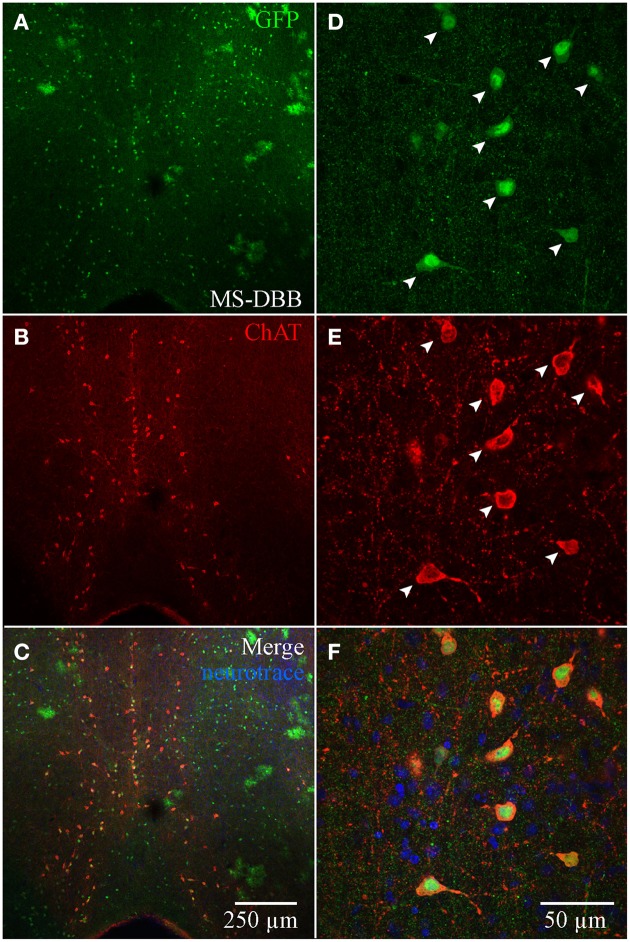
**Anti-ChAT labeling in MS-DBB ChAT-YFP cells. (A)** YFP, **(B)** anti-ChAT, and **(C)** merged images from the MS-DBB. Higher magnification images **(D–F)** showing co-localization of anti-GFP and anti-ChAT labeling. MS-DBB showing co-localization of YFP and ChAT.

**Figure 5:**
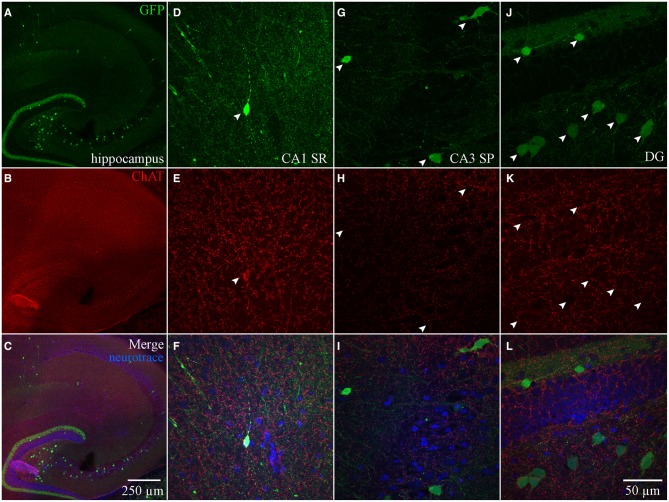
**Anti-ChAT labeling in HC ChAT-YFP cells. (A)** YFP, **(B)** anti-ChAT, and **(C)** merged images from the HC. Higher magnification images in **(D–F)** CA1 SR, **(G–I)** CA3 SP, and **(J–L)** DG showing that anti-ChAT labeling is not detected in ChAT-YFP cells.

Although ChAT has been detected immunocytochemically in the HC, ChAT expression in rat was reported to be weaker in the HC than in the basal forebrain region (Frotscher et al., 1986, 2000). TSA amplification enabled us to achieve high signal-to-noise ratio of ChAT expression (see Material and Methods; Figure S1). In the MS-DBB (Figures 4A–C), ChAT expression was readily detected and co-localized strongly in ChAT-YFP cells (Figures 4A–F). In contrast, despite meticulously processing HC slices in parallel with positive controls in MS-DBB slices, ChAT labeling was observed in ChAT-YFP cells from only HC SR/LM layer (Figures 5A–F), but not in CA3 (Figures 5G–I) or DG (Figures 5J–L). Consistent with findings in ChAT-EGFP mice (von Engelhardt et al., 2007), ChAT expression was only partially overlapping in cortex and striatum (Figures S4A–L).

### A subset of ChAT-YFP cells co-localize with calretinin and VIP

Cortical ChAT-EGFP cells exhibit a high degree of co-localization with the calcium binding protein calretinin and neuropeptide VIP (Bayraktar et al., 1997; von Engelhardt et al., 2007). We sought to examine whether HC ChAT-YFP cells possess a similar degree of co-localization with calretinin and VIP. We found only partial co-localization (8.2%, 146/1785) of calretinin or VIP (2.0%, 22/1088) with ChAT-YFP (Figure S5), with the majority of calretinin-positive ChAT-YFP cells located in CA1 (50.7%, 74/146) and hilus (32.2%, 47/146), and populations sparsely localized to CA3 (15.1%, 22/146) and DG (2.1%, 3/146). In this experiment, some hilar ChAT-YFP neurons were observed to possess spiny proximal dendrites (Figure S5D1, open arrow), suggesting that the diffuse YFP labeling of the DG inner molecular layer (Figure 2A) could be accounted for by the axons of ChAT-YFP-positive mossy cells (Scharfman and Myers, 2012). VIP-positive ChAT-YFP cells were located in CA1 (81.8%, 18/22) and CA3 (18.2%, 4/22). Therefore, our data suggests that there is limited overlap of ChAT-YFP with calretinin and VIP.

### Intrinsic membrane properties of HC ChAT-YFP cells

**Figure 6:**
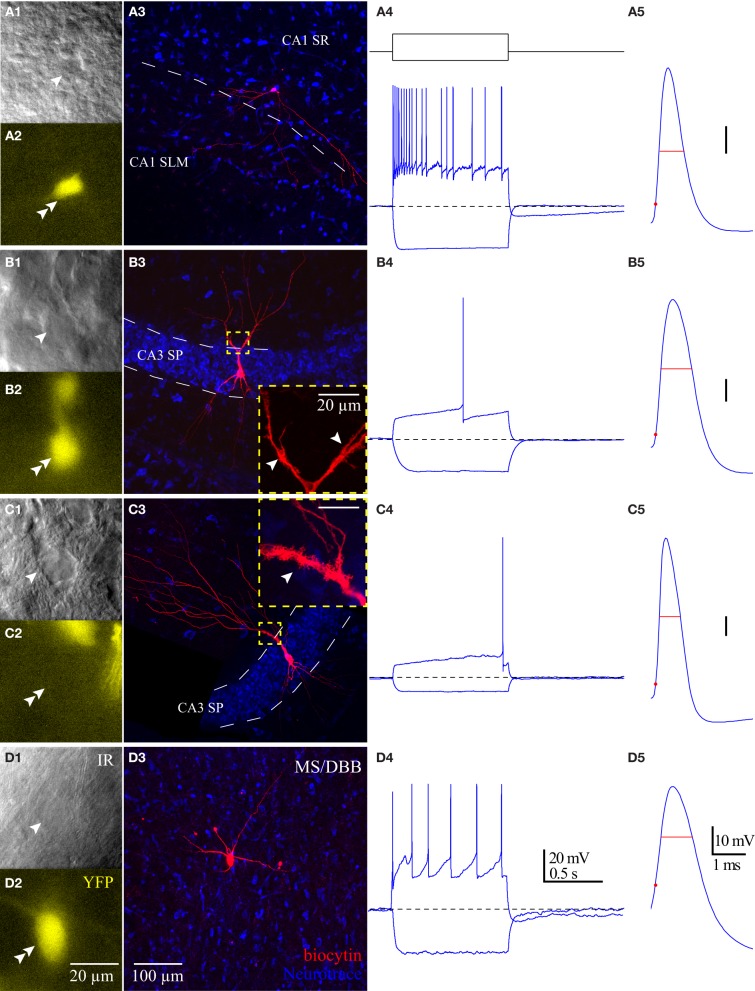
**Properties of HC ChAT-YFP cells**. Morphology and intrinsic membrane properties of representative **(A)** CA1 SR/LM ChAT-YFP, **(B)** CA3 SP ChAT-YFP, **(C)** CA3 SP YFP-negative, and **(D)** MS/DBB ChAT-YFP cells. For each cell type, representative (1) live IR Dodt contrast images, (2) 505 nm fluorescent images, (3) flat-projected confocal image of the biocytin-filled cell, (4) voltage responses to 1 s long hyperpolarizing (−100 pA) or depolarizing (+200 pA) current steps, and (5) first AP half-width are displayed. Insets in panels **(B3,C3)**: thorny excrescences (white arrows) on the dendrites of **(B3)** CA3 SP ChAT-YFP and **(C3)** CA3 SP YFP-negative cells.

**Figure 7:**
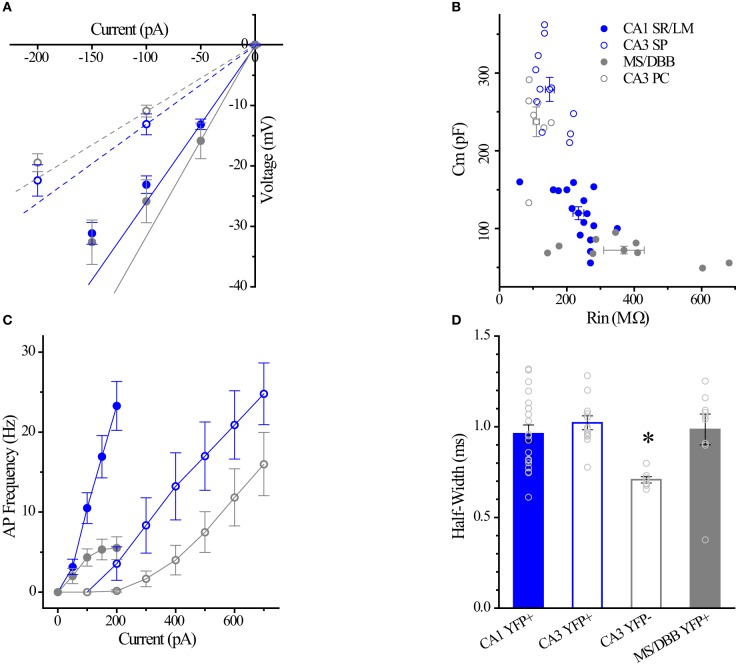
**Differences between CA1 and CA3 ChAT-YFP cells. (A)** I–V relationship for CA1 ChAT-YFP (*n* = 14, blue filled circle), CA3 ChAT-YFP (*n* = 9, blue open circle), CA3 YFP-negative (*n* = 7, gray open circle), and MSDBB ChAT-YFP (*n* = 6, gray filled circle) cells. Lines denote slopes through 0 pA and (solid) −50 pA or (dotted) −100 pA points illustrating differences in R_in_. **(B)** Plot of C_m_ vs. R_in_ for CA1 ChAT-YFP (*n* = 20), CA3 ChAT-YFP (*n* = 12), CA3 YFP-negative (*n* = 7), and MSDBB ChAT-YFP (*n* = 9) cells. **(C)** AP frequency vs. current step for the 4 cell types. **(D)** Bar graph showing narrower half width of CA3 YFP-negative cells than ChAT-YFP cells in CA1 SR/LM, CA3 SP, and MS/DBB (One-Way ANOVA). Asterisk (^*^) denotes *p* < 0.05 compared to CHAT-YFP cells in CA1, CA3 and MS-DBB.

Using whole-cell patch clamp recording from ChAT-Rosa mice, we investigated the intrinsic membrane properties of two populations of HC ChAT-YFP cells in CA1 SR/LM and CA3 SP. Representative live images and morphologies of CA1 SR/LM (Figure 6A) and CA3 SP (Figure 6B) ChAT-YFP cells are shown. As a population, a subset of recorded HC CA1 SR/LM ChAT-YFP cells (5/11, Table 3) exhibited spontaneous firing, which was uncommon in CA3 SP ChAT-YFP cells (1/14). Upon injection of a 1 s long, +200 pA depolarizing current step, ChAT-YFP cells in CA1 SR/LM exhibited an irregular firing pattern (Figure 6A4), while CA3 SP ChAT-YFP cells exhibited a delayed firing phenotype (Figure 6B4). Although AP half-widths were comparable between CA1 SR/LM and CA3 SP ChAT-YFP cells (Figures 6A5,B5, 7D), CA1 SR/LM ChAT-YFP cells had higher R_in_ (Figure 7A) and smaller size (Figure 7B) than CA3 SP ChAT-YFP cells (Table 3). As expected from the differential R_in_ of these populations, CA1 SR/LM ChAT-YFP cells achieved a higher AP frequency than CA3 SP ChAT-YFP cells at small depolarizing current steps (*p* < 0.05 at +100 pA; Table 3), whereas CA3 SP ChAT-YFP cells tolerated larger current steps (Figure 7C). Therefore, the intrinsic membrane properties are distinct between CA1 SR/LM and CA3 SP ChAT-YFP populations, representing distinct ChAT-YFP subclasses.

**Table 3:** **Properties of hippocampal ChAT-YFP neurons**.

**Property**	**CA1 ChAT-YFP**	**CA3 ChAT-YFP**	**CA3 PC**	**MS-DBB YFP**
R_in_ (MΩ)	236.2 ± 13.8^†^^§^^*^	143.8 ± 12.9^§^	125.3 ± 11.3	369.8 ± 60.0^*^
τ_m_ (ms)	25.3 ± 1.8	38.3 ± 3.2	30.3 ± 2.3	25.4 ± 3.2
C_m_ (pF)	111.6 ± 7.6^†^^*^	267.0 ± 16.9^§^	243.2 ± 21.6	72.2 ± 4.8^*^
V_m_ (mV)	−57.9 ± 3.3	−67.9 ± 2.5	−75.0 ± 1.4	−46.9 ± 5.5
AP half width (μs)	969.6 ± 47.8^*^	1021.7 ± 38.0^*^	725.8 ± 26.5	1081.0 ± 42.0^*^
Sag (SS/peak)	0.95 ± 0.01	0.99 ± 0.01	1.00 ± 0.02	0.98 ± 0.01
Spontaneous firing	5/11	1/12	0/7	5/6
AP frequency (at 100 pA)	10.5 ± 1.9	0	0	4.3 ± 1.1

† *Compared to CA3 SP ChAT-YFP cells; p < 0.05*.

§ *Compared to MSDBB ChAT-YFP cells; p < 0.05*.

* *Compared to CA3 SP YFP-negative cells; p < 0.05*.

The intrinsic membrane properties and appearance of thorny excrescences on the apical dendrites of CA3 ChAT-YFP cells (Figure 6B3) led us to compare ChAT-YFP cells to YFP-negative, presumably pyramidal cells, in the CA3 SP (Figure 6C). YFP-negative and ChAT-YFP cells in CA3 SP were not significantly different in C_m_, R_in_, and τ_m_ (Table 3). However, as a population, ChAT-YFP cells had a broader AP half-width than CA3 SP YFP-negative cells (*p* = 0.013; Figures 6B5, 7D). Therefore, on the basis of these findings, CA3 SP ChAT-YFP cells could be viewed as a distinct subclass of CA3 pyramidal cells.

Finally, we examined the intrinsic properties of MS-DBB and HC ChAT-YFP cells. MS-DBB ChAT-YFP cells (Figure 6D) were comparable to CA1 SR/LM cells in C_m_, R_in_, and τ_m_ (Table 3). In addition, MS-DBB ChAT-YFP cells had broad AP half-widths that were not significantly different than AP half-widths in HC ChAT-YFP cells (Figures 6D, 7D; Table 3).

### HC ChAT-YFP cells undergo cholinergic neuromodulation

**Figure 8:**
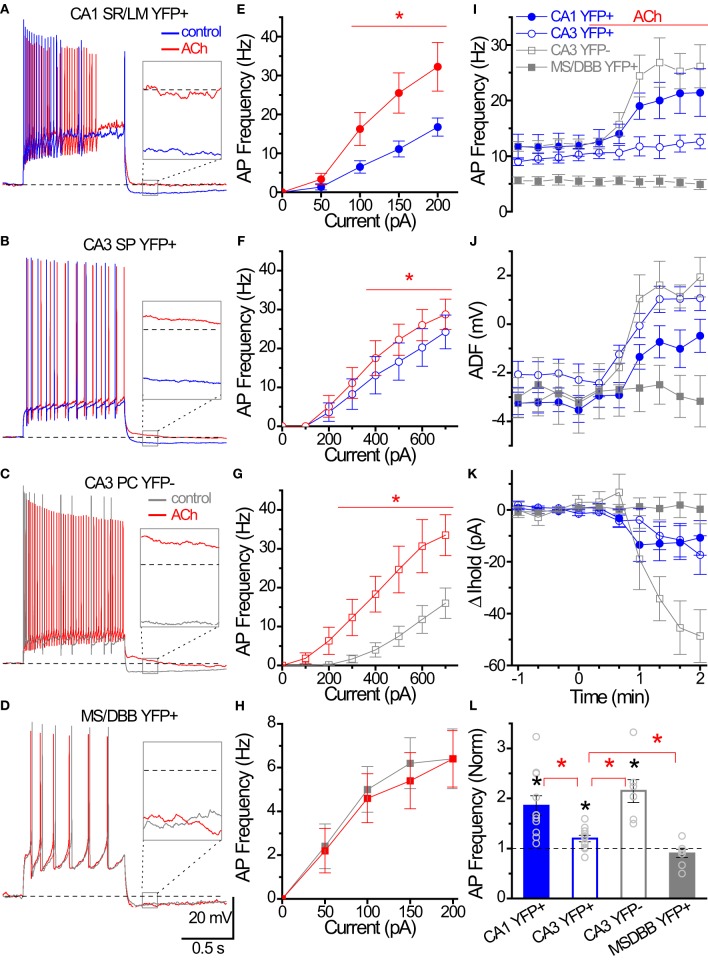
**Cholinergic modulation of Hippocampal ChAT-YFP cells. (A–D)** Whole-cell voltage responses to the introduction of a 1 s long depolarizing current step from −60 mV in representative cells in (blue/gray) control and (red) 100 μM ACh. Inset, solid box expanded to illustrate the effect of ACh on afterdeflection (ADF) (dotted line indicates −60 mV baseline). **(E–H)** Input-output relationships from AP frequency vs. current step magnitude for CA1 YFP+ (E, close circle *n* = 8), and CA3 YFP+ (F, open circle, *n* = 9), CA3 YFP− (G, open square, *n* = 6) and MSDBB YFP+ (H, close square, *n* = 5) cells. ^*^Denotes *p* < 0.05, Two-Way ANOVA. **(I–K)** Population data for the four group cells summarizing the time course of **(I)** AP frequency, **(J)** afterdeflection, **(K)** relative change in I_*hold*_ from (blue) control to (red; at time 0) ACh conditions. **(L)** Bar graph showing cholinergic modulation induced changed in normalized AP frequency. Black asterisks denote *p* < 0.05 compared to 1, one sample *t*-test. Red asterisks denote *p* < 0.05 between groups, One-Way ANOVA.

One intriguing hypothesis is that cholinergic interneurons themselves could be targets of cholinergic modulation (Tricoire and Cea-Del Rio, 2007). To investigate whether ChAT-YFP cells underwent cholinergic modulation, we applied ACh to HC ChAT-YFP cells (Figure 8). In the continuous presence of the AMPA receptor antagonist DNQX (25 μM), the NMDA receptor antagonist APV (50 μM) and the GABAA receptor antagonist gabazine (5 μM), we applied 1 s long depolarizing current to monitor the AP firing frequency of ChAT-YFP cells from CA1 SR/LM ChAT-YFP (Figure 8A) and CA3 SP ChAT-YFP cells (Figure 8B) while introducing bias current to maintain the membrane potential at −60 mV. Bath application of ACh (100 μM) increased AP frequency in CA1 SR/LM ChAT-YFP (100–200 pA step, Figures 8A,B,I) and CA3 SP ChAT-YFP (400–700 pA step, Figures 8C,D,I) cells. In CA1 SR/LM ChAT-YFP cells, the ACh-induced increase in AP frequency (from 13.2 ± 1.8 to 22.8 ± 2.6 Hz, *p* = 0.0007, *n* = 11, two-tailed paired *t*-test; Figure 8I) was accompanied by the elimination of the ADF (from −3.3 ± 0.4 to −0.7 mV ± 0.7 mV, *p* = 0.0029, Wilcoxon matched pairs-signed rank test; Figure 8J) and a modest increase in holding current (by −12.1 ± 6.6 pA, *p* < 0.001, Wilcoxon signed rank test; Figure 8K). In CA3 SP ChAT-YFP cells, the ACh-induced increase in AP frequency (from 10.1 ± 0.8 to 12.1 ± 1.4 Hz, *p* = 0.020, *n* = 11, Wilcoxon matched pairs signed rank test) was more modest and underwent a similar increase in holding current (by −13.0 ± 6.2 pA, *p* < 0.001, Wilcoxon signed rank test), but the AHP was converted to an ADP (from −2.1 ± 0.6 to 1.0 ± 0.5 mV, *p* = 0.002, Wilcoxon matched pairs signed rank test). Similar to previous studies in HC principal cells (Cole and Nicoll, 1983; Cobb and Davies, 2004), application of ACh increased AP frequency (from 12.1 ± 0.9 Hz to 26.1 ± 3.7 Hz, *p* = 0.0044, *n* = 7, paired *t*-test; Figures 8F,I), generated an ADP (−3.0 ± 1.4 to 0.6 ± 0.6 mV, *p* = 0.0003, paired *t*-test; Figure 8J), and increased holding current (−42.8 ± 9.0 pA, *p* = 0.0032, one sample *t*-test; Figure 8K) in CA3 pyramidal cells (PCs). However, the extent that ACh increased AP frequency was larger in CA1 SR/LM ChAT-YFP and CA3 PCs than CA3 SP ChAT-YFP cells (*p* < 0.05, One-Way ANOVA, Figure 8L). Finally, in contrast to HC ChAT-YFP cells, MS-DBB ChAT-YFP cells were not modulated by ACh (*p* > 0.05; Figures 8D,H–L). Taken together, ACh resulted in enhanced ChAT-YFP cells in both CA1 and CA3, but to different extents.

### Optogenetic stimulation of HC ChAT-CRE neurons induces glutamate release onto CA1 interneurons

**Figure 9:**
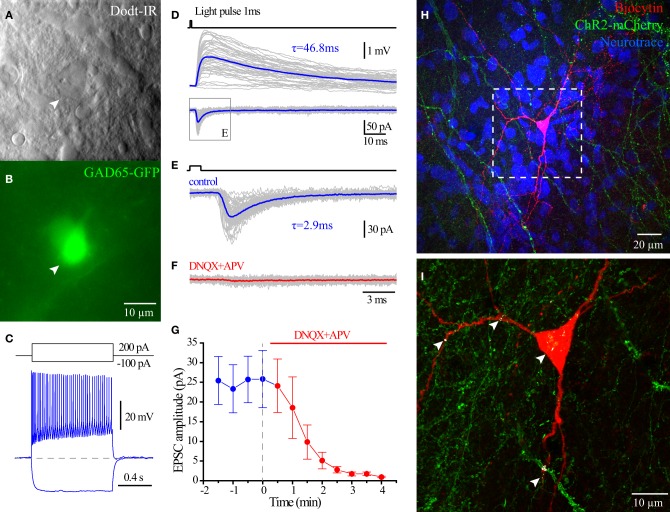
**Glutamate release from hippocampal ChAT-CRE cells**. Live IR Dodt contrast image **(A)** and green fluorescent image **(B)** of a recorded GFP+ cell in hippocampal CA1 SR/LM layer. **(C)** Voltage responses to 1 s long hyperpolarizing (−100 pA) or depolarizing (200 pA) current steps. **(D)** Averaged light flashes (1 ms, 470 nm) induced EPSCs in acsf (blue) and DNQX+APV (red). **(E)** Population date showing the light induced EPSCs were blocked by ionotropic glutamate receptors blockers (Wilcoxon matched-pairs signed rank test, *n* = 6). **(F)** Flat-projection of a confocal image for the biocytin filled (red) cell with mCherry (green) and Neurotrace (blue). **(G)** White regions showing the co-localization of mCherry (ChAT-CRE-positive synaptic terminals) and biocytin (recorded GAD65-GFP cell).

To investigate the neurotransmitter phenotype of HC ChAT-CRE cells, we employed GAD65-GFP/ChAT-CRE mice (see Material and Methods), which enabled optogenetic stimulation of ChAT-CRE cells onto CA1 GAD65-GFP interneurons (López-Bendito et al., 2004; Cea-del Rio et al., 2010; Wierenga et al., 2010). Four weeks after the injection of floxed ChR2-mCherry AAV into HC of GAD65-GFP/ChAT-CRE mice, we performed whole cell recording on GAD65-GFP cells of CA1 SR/SLM (Figures 9A–C). In response to 470 nm light flashes (1–5 ms), EPSCs were evoked in 7/17 of recorded CA1 SR/SLM GAD65-GFP cells. Perfusion of AMPA and NMDA receptor blockers DNQX and APV fully blocked the light pulses induced EPSCs in 6/7 cells (Figures 9D,E). Consistent with monosynaptic stimulation, the onset of the glutamate EPSC occurred ~2 ms after onset of light stimulation. Post-hoc confocal analysis revealed colocalization points of ChR2-mCherry-positive presynaptic terminals with the somatodendritc region of recorded GAD65-GFP cells (Figures 9H,I), consistent with direct synaptic input from ChAT-CRE cells. In 1/7 cells, optogenetic stimulation elicited an inward polysynaptic current that was resistant to block by DNQX, APV, and mAChR antagonist atropine. Overall, these data indicate that optogenetic stimulation of intrinsic HC ChAT-CRE neurons results in glutamatergic excitation.

## Discussion

### Neurochemical identity and cellular distribution of HC neurons visualized in ChAT-tauGFP and ChAT-Rosa mice

HC cholinergic interneurons were described almost 30 years ago (Frotscher et al., 1986), and, in modern HC interneuron classification schemes, are recognized as one of over 21 distinct HC interneuron subtypes (Klausberger and Somogyi, 2008). In a previous study, ChAT-expressing neurons were shown to lack GAD65/67 mRNA (Frotscher et al., 2000). However, in cortex, ChAT-EGFP bipolar neurons immunoreactive for ChAT co-localize strongly with VIP and calretinin (von Engelhardt et al., 2007), which could overlap with GABAergic interneuron subtypes (Chamberland et al., 2010; Chamberland and Topolnik, 2012; Tyan et al., 2014). By contrast, cortical bipolar ChAT-EGFP cells lack GAD67 mRNA (von Engelhardt et al., 2007). Moreover, VIP/calretinin-positive interneurons tended to exhibit an irregular spiking, or stuttering phenotype (Porter et al., 1999; von Engelhardt et al., 2007).

In the present study, we investigated HC neurons that expressed fluorescent proteins under the control of the ChAT promoter (Gong et al., 2007; Grybko et al., 2011), enabling HC ChAT-positive cells to be revealed through transgenic mouse technology. In the initial examination of the HC of ChAT-tauGFP mice, GFP-containing fibers were found to densely innervate all layers of the hippocampus, most of which presumably arose from MS-DBB cholinergic projection neurons (Dutar et al., 1995). Although our use of Neurotrace was originally intended only to define HC layers, upon higher magnification, we noted that some tauGFP-positive structures that were originally counted as cells were actually negative for Neurotrace (Figure S3). We then used both Neurotrace and DAPI as cellular markers to unambiguously identify HC neurons in the ChAT-tauGFP population (Figure 1; Table 1). Consistent with HC ChAT-immunoreactive interneurons in rat (Frotscher et al., 1986, 2000) and ChAT-EGFP neurons in mice (von Engelhardt and colleagues, unpublished observations), we revealed that 59.4% of ChAT-tauGFP structures were labeled with both Neurotrace and DAPI (Figure 3A), particularly in CA1 and CA3 SR/SLM regions (Figure 3C). Interestingly, 40.6% of ChAT-tauGFP structures, which were comparable in diameter to the neuronal ChAT-tauGFP population (Figures 3A,B), lacked Neurotrace and DAPI (Figure 3A). These ChAT-tauGFP structures tended to be found in CA1 and CA3 stratum oriens, which may be a reflection of the density of MS-DBB cholinergic afferents in HC layers. Axon blebs, which can approach the size of ChAT-tauGFP globular structures measured here, are observed only after the axon is severed (Shu et al., 2006; Hu and Shu, 2012). However, the ChAT-tauGFP axon appears to be intact (Figure 1K, inset), resembling large, en passant boutons. These globular structures could result from the overexpression of tauGFP in cholinergic axons, and are therefore specific to ChAT-tauGFP mice. Supporting this hypothesis, cell sized ChAT-YFP-positive structures were invariably Neurotrace-positive neurons in ChAT-Rosa mice. In any case, the large diameter of many of these boutons provides a future opportunity to access cholinergic axons electrophysiologically and understand their intrinsic membrane properties, firing activity, and presynaptic modulatory capacity.

The GM24 line of ChAT-CRE mice generally has been used in combination with a ChR2 AAV or with a reporter line to optically stimulate (Witten et al., 2010) and/or visualize cholinergic neurons (Ivanova et al., 2010; Lopes et al., 2012) in various brain regions. However, no study has yet examined YFP-positive cells in the hippocampus of ChAT-Rosa mice. In examining the regional and laminar distribution of ChAT-YFP cells (Figure 2), there was some similarity to the original description of ChAT cells in the HC (Frotscher et al., 1986), especially in the location of small cells in the CA1 SR/SLM layer (Figures 2A,G). A majority (15/19) of CA1 SR/SLM neurons possessed a stuttering/irregular firing phenotype (Figure 6), which is reminiscent of the phenotypes of cortical ChAT-EGFP (von Engelhardt et al., 2007), cortical VIP/calretinin/ChAT (Porter et al., 1998, 1999), and HC VIP/calretinin (Tyan et al., 2014) interneuron subtypes.

In addition to similarities, we noted several differences when comparing the cellular distribution and neurochemical identity of ChAT-YFP cells with previous studies, as well as with the ChAT-tauGFP population also described here. Despite the strong co-localization of ChAT immunoreactivity in MS-DBB ChAT-YFP cells processed in parallel, ChAT immunoreactivity was rarely detected in HC ChAT-YFP cells. There are a number of possibilities that could explain this discrepancy. First, the discrepancy between YFP and anti-ChAT signals could be due to the ectopic expression of CRE in non-ChAT cells (Gong et al., 2007). Second, ChAT immunoreactivity is weaker in the hippocampus (Frotscher et al., 1986) and cortex (von Engelhardt et al., 2007) than in basal forebrain. Driving EGFP or EYFP expression under the control of the ChAT promoter may amplify the detection sensitivity of ChAT-expressing cells above the detection sensitivity of the anti-ChAT antibody. Third, access of the anti-ChAT antibody to the epitope binding site on ChAT may differ depending on the region and/or cell type. Fourth, CRE/loxP recombination may temporally dissociate YFP expression from ChAT expression. Unlike ChAT-EGFP (von Engelhardt et al., 2007) and ChAT-tauGFP (Grybko et al., 2011) mice, where ChAT and GFP expression are expected to be temporally correlated (Erickson et al., 2014), YFP expression in a ChAT-YFP neuron may only indicate that ChAT was transiently expressed during development of the ChAT-YFP neuron. Therefore, YFP may be expressed even if ChAT expression was strongly down-regulated during development.

In a recent study, fetal HC neurons immunopositive for vesicular glutamate transporter 1 (vGluT1) were observed to co-localize with ChAT (Bhargava et al., 2010), consistent with previous observations demonstrating glutamate and ACh co-release in some types of cholinergic neurons (Allen et al., 2006; Ren et al., 2011). However, ChAT immunoreactivity was absent from all but a small subpopulation of vGluT1-positive neonatal cells grown for 13 days in culture (Bhargava et al., 2010). Consistent with the idea that ChAT expression is downregulated in glutamatergic neurons, we found that a subset of CA3 SP neurons express ChAT-YFP (Figures 2A,F) but do not exhibit ChAT immunoreactivity (Figures 5A–C, G–I). Moreover, CA3 SP neurons were not well represented in ChAT-tauGFP mice (Figure 3C). However, optogenetic stimulation induced glutamatergic EPSCs in CA1 HC of ChAT-CRE mice (Figures 9D–G), indicating that CRE expression must persist in adult ChAT-CRE mice. ChR2-mCherry-expressing synaptic terminals were observed in CA1 (Figures 9H,I), most likely originating from CA3 SP ChAT-CRE cells. Although ChAT-YFP cells in the CA3 area that resemble pyramidal cells are distinct from non-fluorescent pyramidal cells (Figure 7D), these observations are consistent with conventional glutamatergic transmission from CA3 ChAT-CRE cells. CA3 ChAT-CRE cells most likely misexpress CRE-recombinase because they have no equivalent in the ChAT-tau GFP mice and exhibit no detectable ChAT immunoreactivity. In hilar ChAT-YFP cells, the presence of thorny excrescences (Figure S5D) and the diffuse YFP labeling in the DG inner molecular layer (Figure S5A) suggest that at least some of ChAT-YFP cells in hilus are mossy cells (Scharfman and Myers, 2012). Given the above observations, it will be of interest to examine other ChAT-CRE mouse lines for the presence of these cell populations.

Sparse ChAT immunoreactivity and some overlap with calretinin/VIP was observed in some CA1 cells, consistent with a population of Neurotrace-positive CA1 SR/SLM interneurons in ChAT-tauGFP mice (Figure 3C). These observations are consistent with the retention of ChAT, albeit at a lower level than in MS-DBB cholinergic neurons, in a small subset of the adult CA1 SR/SLM interneuron population (Figures 4, 5). A subset of CA1 VIP/calretinin interneurons are clearly GABAergic (Chamberland et al., 2010; Chamberland and Topolnik, 2012; Tyan et al., 2014), consistent with original observations made in cortex (Bayraktar et al., 1997; von Engelhardt et al., 2007). However, in situ hybridization data showed lack of GAD65/67 mRNA expression in rat ChAT immunopositive HC interneurons (Frotscher et al., 2000). We attempted to optogentically stimulate this CA1 interneuron population; however, it is likely that the glutamatergic output from CRE-expressing principal cells in CA3 or DG confounded this experimental design. Although it is possible that glutamate is also released (or co-released) from ChAT-CRE cells in CA1 or CA3 SR/SLM, future experiments that more narrowly focus on optogenetic stimulation of this population would unambiguously reveal neurotransmitter phenotype(s) of this interneuron subclass.

### Cholinergic modulation of “cholinergic interneurons” during HC network operations

ACh release is associated with HC-related behaviors (Pepeu and Giovannini, 2004). In the present study, we found that bath application of ACh enhanced the excitability of both HC CA1 SR/SLM and CA3 ChAT-YFP cells. Although we did not differentiate between nicotinic and muscarinic activation, our results clearly demonstrate that the cellular excitability of ChAT-YFP neurons is influenced by cholinergic neuromodulation. The ACh-induced increase in cellular excitability is most likely due to the activation of mAChRs, as suggested by similarities in mAChR-induced changes in intrinsic membrane properties similar to previous studies (Cole and Nicoll, 1983; Lawrence et al., 2006; Cea-del Rio et al., 2010; Dasari and Gulledge, 2011). However, it is possible that a subset of CA1 SR/SLM ChAT-YFP cells are enriched with a high density of nAChRs, as found in calretinin/VIP-positive cells in cortex (Porter et al., 1999). Indeed, a subset of ChAT-tauGFP axons arising from Neurotrace/DAPI-positive ChAT-tauGFP HC interneurons may contribute to the generation of α7 nAChR-mediated EPSCs in CA3 pyramidal cells (Grybko et al., 2011). Nevertheless, these observations raise the possibility that cholinergic interneurons could be excited by the endogenous release of ACh arising from either extrinsic or intrinsic sources.

What are the potential consequences of cholinergic modulation of ChAT-expressing neurons in the HC? Since ACh release from HC ChAT+ cells has not been definitively demonstrated, it remains an unresolved issue, but given the possible developmental downregulation of ChAT expression (Bhargava et al., 2010), ACh release from HC interneurons is likely to play a more prominent role during development than in the adult. Future studies investigating the expression of ChAT immunoreactivity during development of the HC are needed to clarify this issue. It is also possible that these same HC neurons may regress to an earlier stage of development under pathological conditions, and upregulate their synthesis and release of ACh. Evidence for cholinergic dysfunction occurs in epileptic tissue (Romo-Parra et al., 2003), and the abnormal release of ACh may account in part for such dysfunction. Finally, given the partial overlap with VIP and calretinin (Figure S5), we cannot rule out possible roles of ACh release from HC ChAT+ cells in neurovascular and/or neurometabolic coupling (Cauli et al., 2004, 2014).

Taken together, our efforts to investigate HC interneurons through transgenic mouse technology have improved our understanding of this heterogeneous class of neurons. However, many questions remain regarding their neurochemical identity, significance to HC function, and potential interaction with MS-DBB cholinergic networks. Next generation transgenic technology may provide additional tools that can be applied to examine distinct subpopulations of HC cholinergic interneurons.

### Conflict of interest statement

The authors declare that the research was conducted in the absence of any commercial or financial relationships that could be construed as a potential conflict of interest.

## Supplementary material

Figure S1 **Control experiments for anti-ChAT staining**. Primary and secondary antibodies for GFP were added in all the experiments. **(A,B)** MS ChAT cells (red) were not revealed in goat anti-ChAT primary only slices. **(C,D)** MS ChAT cells were not revealed in non-primary incubated slices. **(E,F)** ChAT cells were detected in slices incubated with anti-ChAT primary, secondary and TSA.

Figure S2 **Optogenetic stimulation of HC ChAT-CRE cells. (A)** Live ChR2-mCherry (590 nm) fluorescence and **(B)** live Dodt-IR contrast image of a ChAT-CRE cell in hippocampus CA1 SR layer. **(C)** Voltage responses upon introduction of (shift-option) ± 200 pA current steps. **(D)** Delivery of 470 nm flashes (blue, 2 ms duration) at 5 Hz for 5 s induced APs in the recorded ChR2-mCherry+ cell. Inset: overlaid AP waveforms.

Figure S3 **Presence of HC ChAT-tauGFP cells and structures in ChAT-tauGFP mice. (A)** Flat-projected confocal image displaying (green) ChAT-tauGFP cells in the hippocampus. Cells and layers are counterstained with (blue) Neurotrace 435/455 Blue Fluorescent Nissl Stain. **(B–D)** Magnified views of a ChAT-tauGFP in CA1 SR region. **(E–J)** Large Neurotrace-negative ChAT-tauGFP structures resembling an en passant bouton.

Figure S4 **Detection of ChAT immunoreactivity in cortex and striatum. (A)** YFP, **(B)** anti-ChAT, and **(C)** merged images from the cortex. Higher magnification images in **(D–F)**. **(G)** YFP, **(H)** anti-ChAT, and **(I)** merged images from the striatum. Higher magnification images in **(J–L)**. Arrows indicating GFP cells.

Figure S5 **A subset of HC ChAT-YFP cells co-localize with calretinin and VIP. (A)** Flat-projected confocal image of the HC from a ChAT-Rosa mouse. Immunoreactivity for ChAT-YFP (green), calretinin (CR, blue), and VIP (red) are shown. **(B–E)** Magnified views of a ChAT-YFP cells in CA1 SR **(B)**, CA3 SP **(C)**, hilus **(D)**, and dentate gyrus **(E)**. Filled arrows denote co-localization of CR and/or VIP with ChAT-YFP cells; the open arrow denotes the thorny excrescences on one ChAT-YFP cell in hilus, suggestive of a mossy cell.
