# Supplementary figures and images for: Hippocampal “cholinergic interneurons” visualized with the choline acetyltransferase promoter: anatomical distribution, intrinsic membrane properties, neurochemical characteristics, and capacity for cholinergic modulation
Source: Front Synaptic Neurosci. 2015 Mar 6;7:4. doi: 10.3389/fnsyn.2015.00004 (PMC4351620; doi:10.3389/fnsyn.2015.00004)

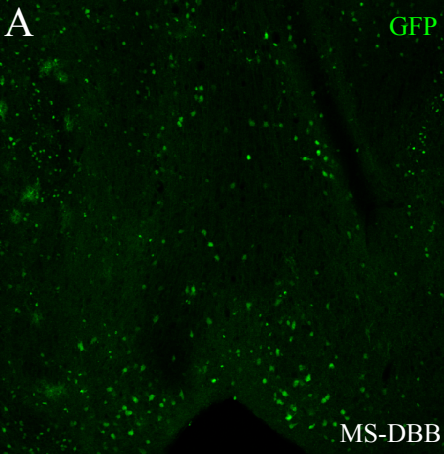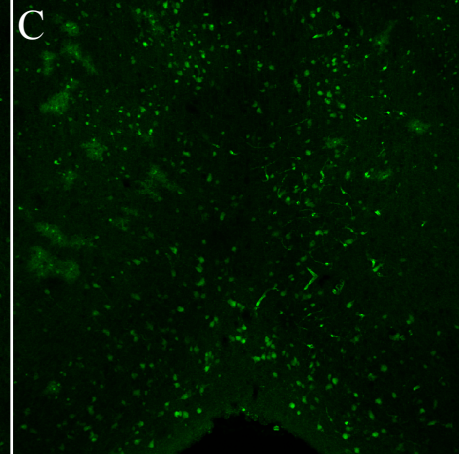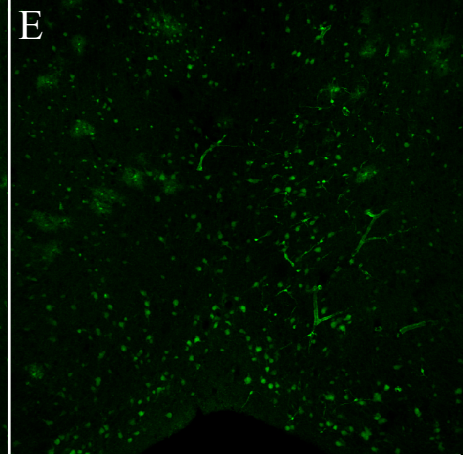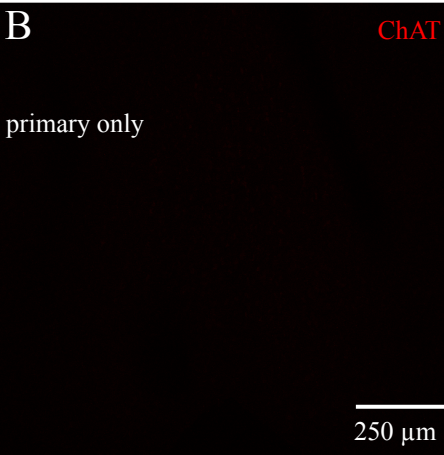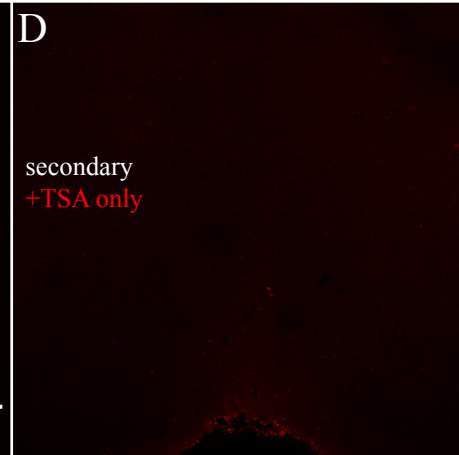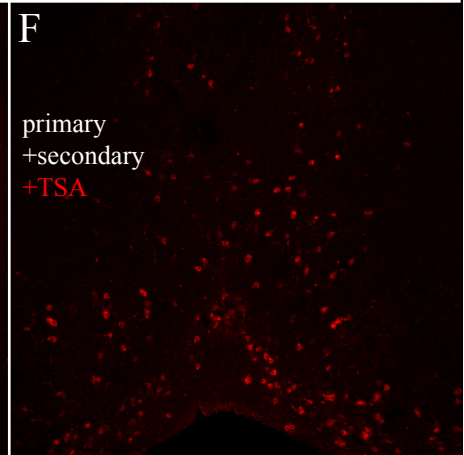

250  $\mu$ m

Supplement: Figure S1 — Control experiments for anti-ChAT staining. Primary and secondary antibodies for GFP were added in all the experiments. (A,B) MS ChAT cells (red) were not revealed in goat anti-ChAT primary only slices. (C,D) MS ChAT cells were not revealed in non-primary incubated slices. (E,F) ChAT cells were detected in slices incubated with anti-ChAT primary, secondary and TSA. [file Image1.PDF]

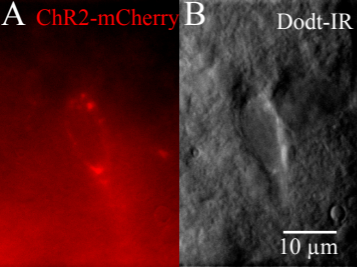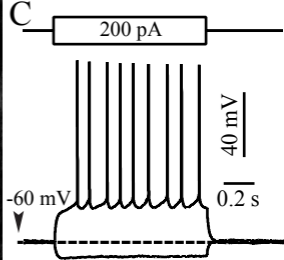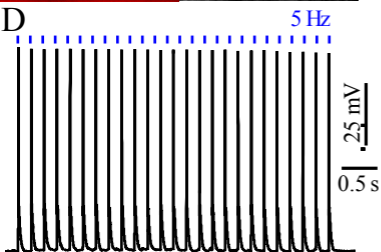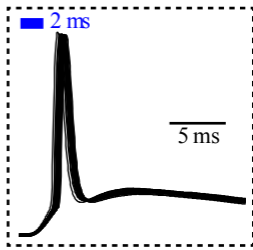

Supplement: Figure S2 — Optogenetic stimulation of HC ChAT-CRE cells. (A) Live ChR2-mCherry (590 nm) fluorescence and (B) live Dodt-IR contrast image of a ChAT-CRE cell in hippocampus CA1 SR layer. (C) Voltage responses upon introduction of (shift-option) ± 200 pA current steps. (D) Delivery of 470 nm flashes (blue, 2 ms duration) at 5 Hz for 5 s induced APs in the recorded ChR2-mCherry+ cell. Inset: overlaid AP waveforms. [file Image2.PDF]

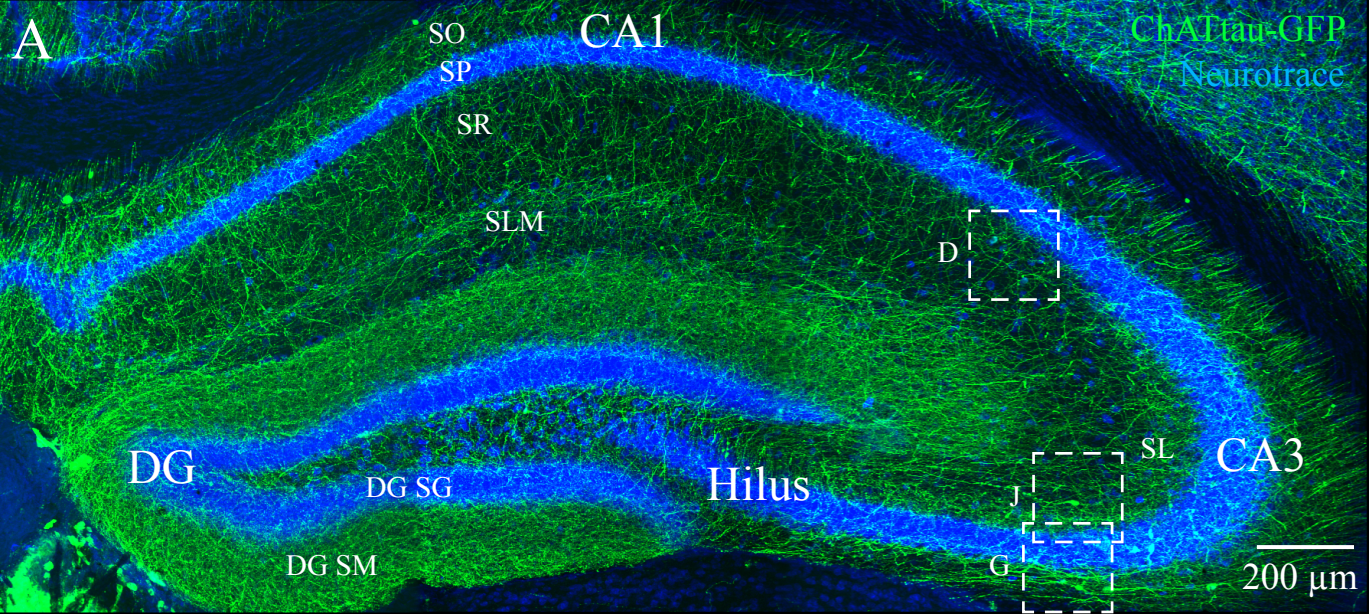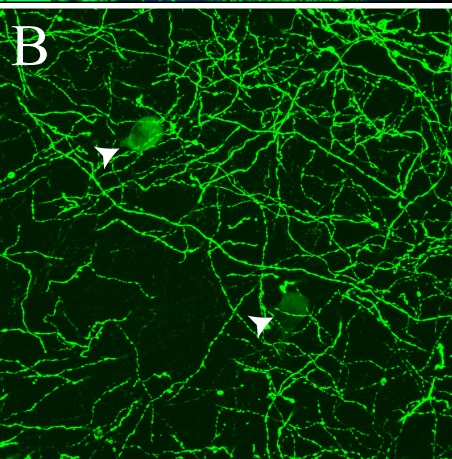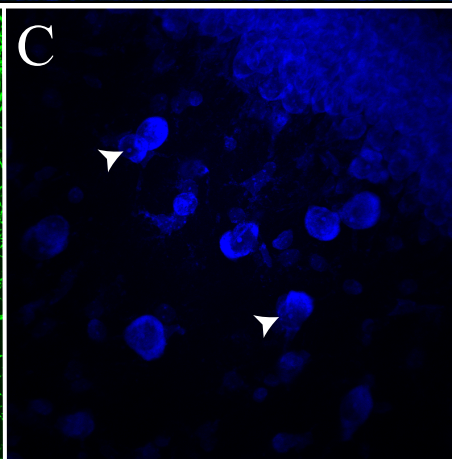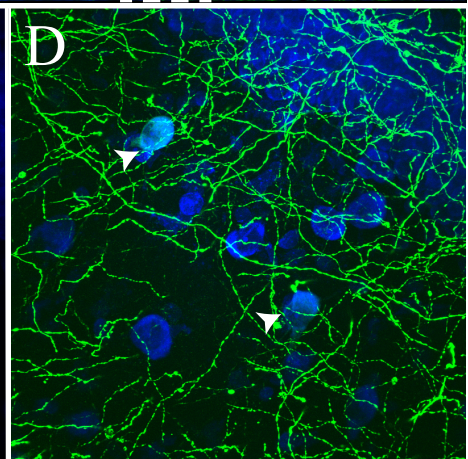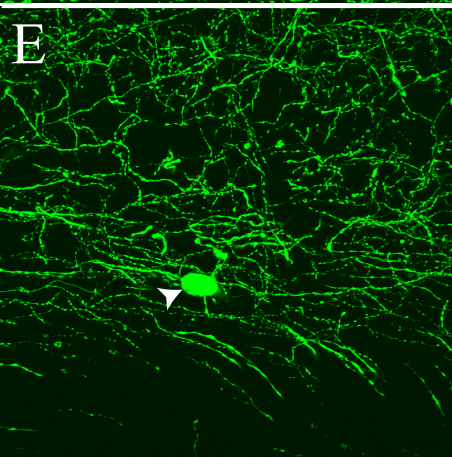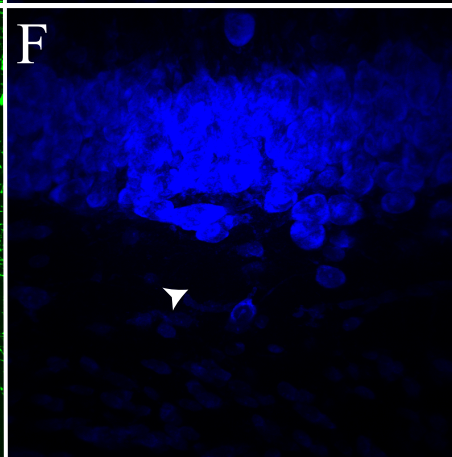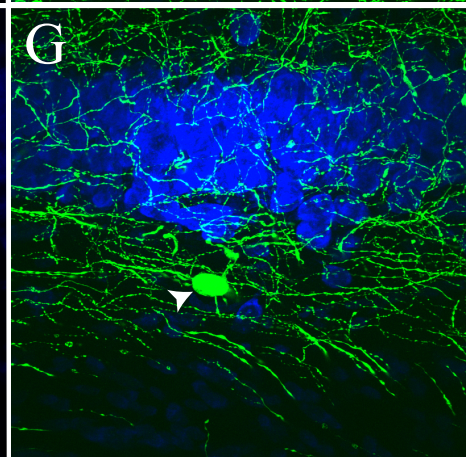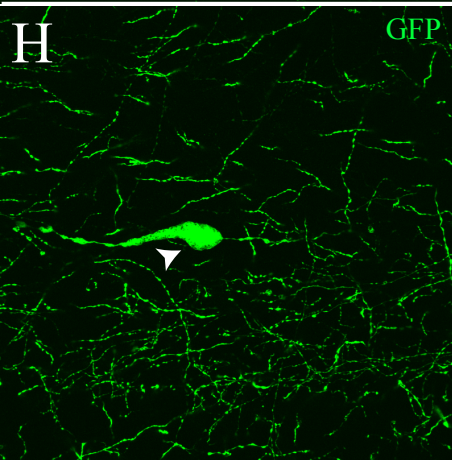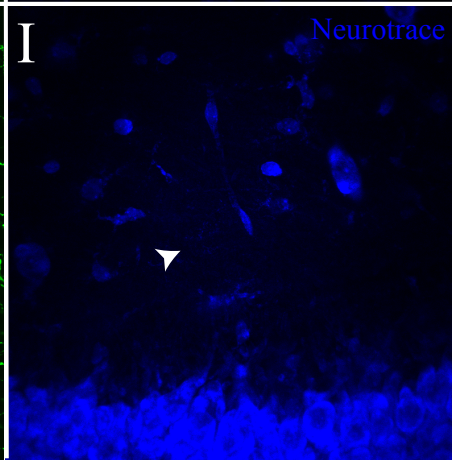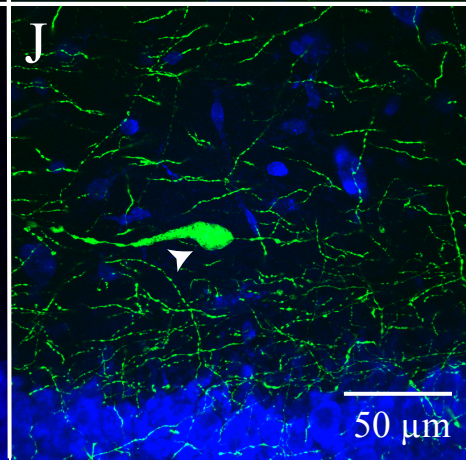

Supplement: Figure S3 — Presence of HC ChAT-tauGFP cells and structures in ChAT-tauGFP mice. (A) Flat-projected confocal image displaying (green) ChAT-tauGFP cells in the hippocampus. Cells and layers are counterstained with (blue) Neurotrace 435/455 Blue Fluorescent Nissl Stain. (B–D) Magnified views of a ChAT-tauGFP in CA1 SR region. (E–J) Large Neurotrace-negative ChAT-tauGFP structures resembling an en passant bouton. [file Image3.PDF]

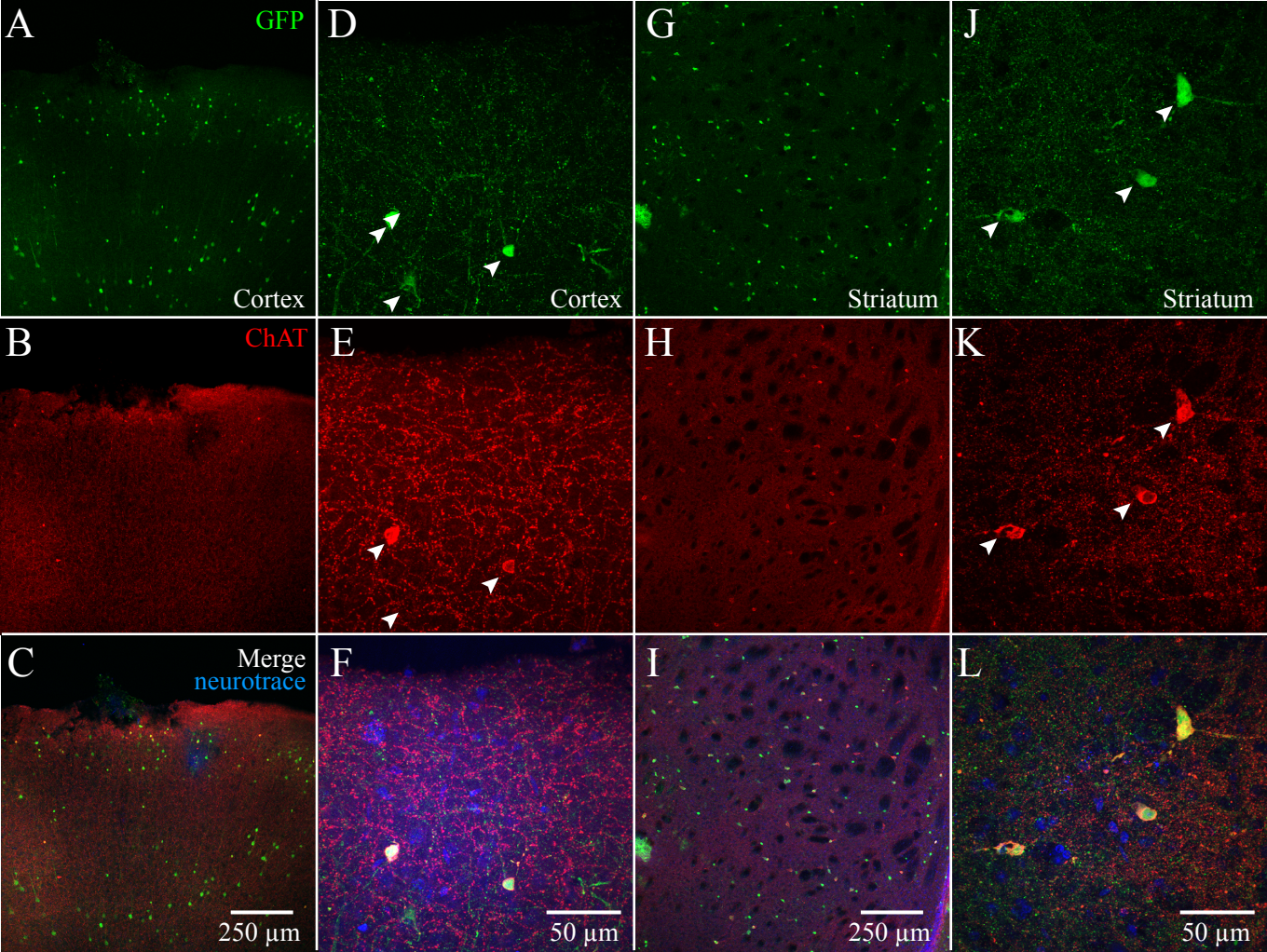

Supplement: Figure S4 — Detection of ChAT immunoreactivity in cortex and striatum. (A) YFP, (B) anti-ChAT, and (C) merged images from the cortex. Higher magnification images in (D–F). (G) YFP, (H) anti-ChAT, and (I) merged images from the striatum. Higher magnification images in (J–L). Arrows indicating GFP cells. [file Image4.PDF]

A

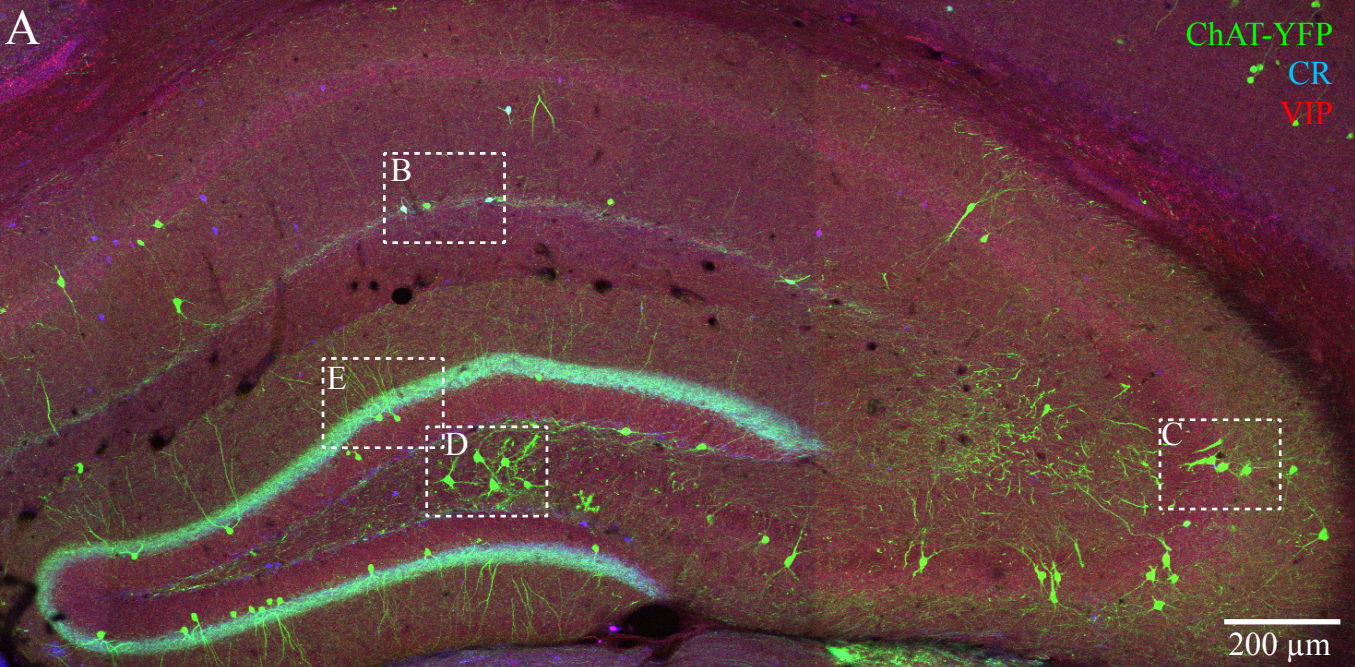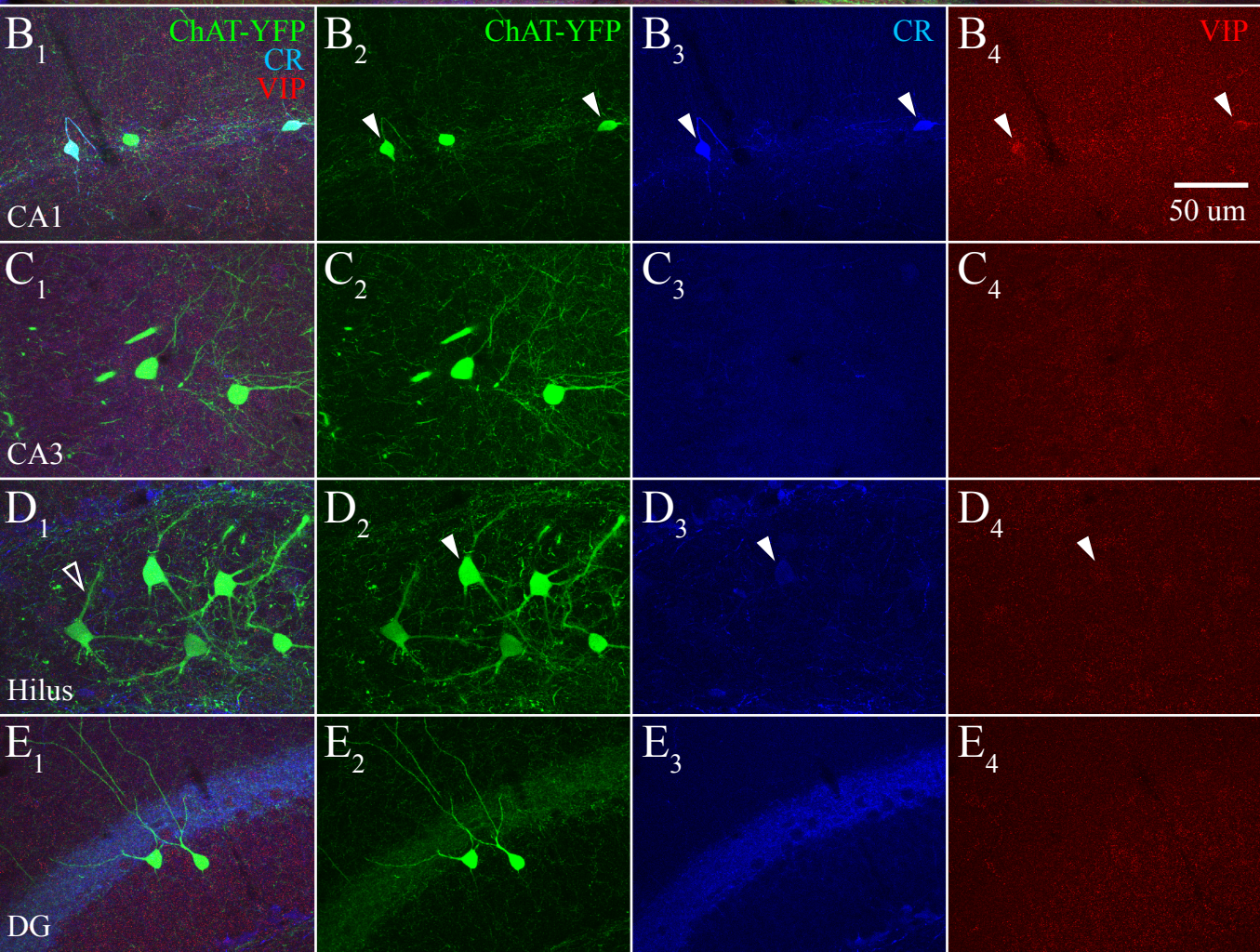

Supplement: Figure S5 — A subset of HC ChAT-YFP cells co-localize with calretinin and VIP. (A) Flat-projected confocal image of the HC from a ChAT-Rosa mouse. Immunoreactivity for ChAT-YFP (green), calretinin (CR, blue), and VIP (red) are shown. (B–E) Magnified views of a ChAT-YFP cells in CA1 SR (B), CA3 SP (C), hilus (D), and dentate gyrus (E). Filled arrows denote co-localization of CR and/or VIP with ChAT-YFP cells; the open arrow denotes the thorny excrescences on one ChAT-YFP cell in hilus, suggestive of a mossy cell. [file Image5.PDF]
